# Mid-winter de-acclimation triggers a stress-like response in leaves of cold-acclimated wheat (*Triticum aestivum* L.) at the tillering stage

**DOI:** 10.1186/s12870-026-09102-8

**Published:** 2026-06-02

**Authors:** Magdalena Wójcik-Jagła, Ipsa Bani, Martin Kovacik, Ales Pecinka, Piotr Waligórski, Marcin Rapacz

**Affiliations:** 1 Department of Plant Breeding, Physiology and Seed Science, University of Agriculture in Krakow, al. Mickiewicza 21, Krakow, 31-120 Poland; 2 Centre of Plant Structural and Functional Genomics, Institute of Experimental Botany, Czech Acad Sci, Šlechtitelů 31, Olomouc, 779 00 Czechia; 3 The Franciszek Górski Institute of Plant Physiology, Polish Academy of Sciences, Niezapominajek 21, Krakow, 30-239 Poland

**Keywords:** De-acclimation, Climate change, Freezing tolerance, Hormone signalling, RNA sequencing

## Abstract

**Background:**

Active de-acclimation, triggered by mid-winter warm spells, threatens winter wheat survival under freezing conditions. This study investigated the physiological and molecular mechanisms underlying the response to active de-acclimation in eight wheat accessions with contrasting tolerance levels.

**Results:**

Phenotyping showed that tolerant accessions maintained high survival rates and photosynthetic efficiency after freezing in the de-acclimated state, whereas the same treatment was lethal to susceptible lines. Chlorophyll fluorescence parameters and principal component analysis clearly separated tolerant from sensitive accessions, indicating distinct genetic bases of freezing tolerance in cold-acclimated and de-acclimated states. RNA sequencing demonstrated that active de-acclimation is not merely the reversal of cold acclimation, as numerous genes were uniquely differentially expressed during this process. Susceptible accessions exhibited broader and more pronounced transcriptomic changes than tolerant ones, suggesting that tolerance involves a more controlled molecular response to warming. Gene ontology analyses showed enrichment of transcripts related to ethylene perception, hormone signalling, root development, and metal ion transport specifically associated with de-acclimation. Expression analysis and hormone profiling highlighted the role of abscisic acid (ABA), salicylic acid (SA), and selected cytokinins in differentiating tolerant and susceptible accessions. Notably, ABA hydroxylase expression and ABA levels were closely associated with tolerance. qPCR validation confirmed RNA-seq results for key hormone-related candidate genes.

**Conclusions:**

Active de-acclimation in wheat is a complex response involving stress-related pathways, particularly hormone signalling. Cold acclimation capacity is critical for subsequent tolerance, and dual selection for freezing and de-acclimation tolerance is recommended in breeding programs. These findings identify ABA- and ethylene-related pathways as key targets for improving resilience to fluctuating winter temperatures.

**Supplementary Information:**

The online version contains supplementary material available at 10.1186/s12870-026-09102-8.

## Background

According to future winter survival scenarios climate change will inevitably lead to decreased winterhardiness in Central Europe [1–3]. The most important factor influencing future winter survival will be mid-winter de-acclimation [3]. De-acclimation is defined as the loss of freezing tolerance during temporary warm periods. Two distinct types of de-acclimation are recognized. Active de-acclimation occurs during winter, when plants should remain cold-acclimated [4]. Passive de-acclimation, by contrast, takes place in spring and is associated with developmental transitions from vegetative to generative stages [1]. This distinction is essential, as these two processes differ both physiologically and molecularly [5]. At the molecular level, active de-acclimation has been shown to be a separate phenomenon in barley plants, not simply the reverse of cold-acclimation [6].

Tolerance to de-acclimation amounts to freezing tolerance after a period of warming [2, 5]. If, after a period of warming, temperatures decrease gradually, plants may reacclimate [5, 6]. On the contrary, if high temperatures persist for a longer period, plants will enter a mode of passive de-acclimation and developmental changes [7]. Therefore, to understand the mechanisms underlying tolerance to active de-acclimation, it is necessary to analyse plant responses during short-term warming events together with monitoring changes in freezing tolerance.

In addition to cereals like wheat and barley (*Hordeum vulgare* L.), tolerance to de-acclimation has been extensively studied in crucifer dicots, e.g. oilseed rape, and *Arabidopsis thaliana* [5, 6, 8, 9]. Studies in barley have demonstrated that tolerance to de-acclimation is influenced by mutations in the *HvDWARF*, *HvCPD*, and *HvBR11* genes. These mutations impact the signalling and biosynthesis of brassinosteroids (BR) [10]. Disturbances in phytohormone levels affect freezing tolerance after de-acclimation [8]. Importantly, de-acclimation tolerance is independent of freezing tolerance, with the photosynthetic apparatus playing a key role in both processes. Therefore, chlorophyll fluorescence parameters, particularly ET_0_/CS, were found to be a useful indicator of tolerance to de-acclimation [5]. Moreover, recent studies revealed that microRNAs (miRNAs) play a crucial role in regulating various physiological processes, including plant responses to cold stress. In barley, several novel miRNAs and their corresponding target genes have been identified as involved in cold de-acclimation, providing promising targets for improving tolerance [11]. Similarly, it was found that tolerance to de-acclimation in oilseed rape was cultivar-dependent [8]. Changes in the water band index (WBI) after de-acclimation may indicate both improved adjustment to higher temperatures and reduced tolerance to de-acclimation [12]. Furthermore, other studies demonstrated that Arabidopsis tolerance to de-acclimation arises from natural variations in de-acclimation rates, partly regulated by the plastid antioxidant system [13].

The rate of de-acclimation is influenced by several factors, with temperature, duration of the warm period, and light conditions being the most critical [7]. Generally, higher temperatures and prolonged exposure to warmth accelerate de-acclimation [9]. Different treatments used in de-acclimation studies involve varying durations and environmental parameters that significantly affect plant physiological responses. A study in *Arabidopsis thaliana* reported a sharp decline in freezing tolerance within three days after transfer to ambient temperatures [14]. Similarly, a seven-day de-acclimation period resulted in significant changes in metabolic accumulation [15]. Temperature fluctuations between day and night also play a role, with higher night temperatures being particularly important for effective de-acclimation [9]. Previous research has also shown that the rising global temperatures have made de-acclimation a critical determinant of winter survival in cereals [16]. However, many studies use conditions more characteristic of passive than active de-acclimation. This limits their relevance to mid-winter warming events. To realistically simulate the field conditions, our de-acclimation treatments were designed based on the temperature patterns, light regimes, and duration of warm periods typical of Central European winters [17, 18].

It has been found that winter wheat lines bred in Poland show a significant degree of variation in tolerance to mid-winter de-acclimation [16]. Previous hypotheses suggest that this variation may be related to differences in reactive oxygen species dynamics and antioxidant activity. During warming, reduced ROS production and more efficient antioxidant activity may contribute to de-acclimation. Tolerant genotypes may exhibit a reduced sensitivity to these changes [4].

So far, breeding programmes specifically targeting active de-acclimation tolerance in wheat have not been conducted in Poland. However, previous studies suggest that indirect selection for this trait may have occurred [3, 5]. The diversity of Polish advanced wheat breeding lines in terms of de-acclimation tolerance is significant [16]. This enables the selection of highly tolerant and highly sensitive forms. However, the physiological and molecular mechanisms underlying tolerance to active de-acclimation in wheat remain insufficiently understood.

Therefore, the aim of this study was to identify physiological and genetic mechanisms of tolerance to active de-acclimation in winter wheat by comparing four tolerant and four susceptible accessions (cultivars and advanced breeding lines).

## Results

### Freezing tolerance in cold-acclimated state and freezing tolerance in de-acclimated state are independent traits

**Fig. 1:**
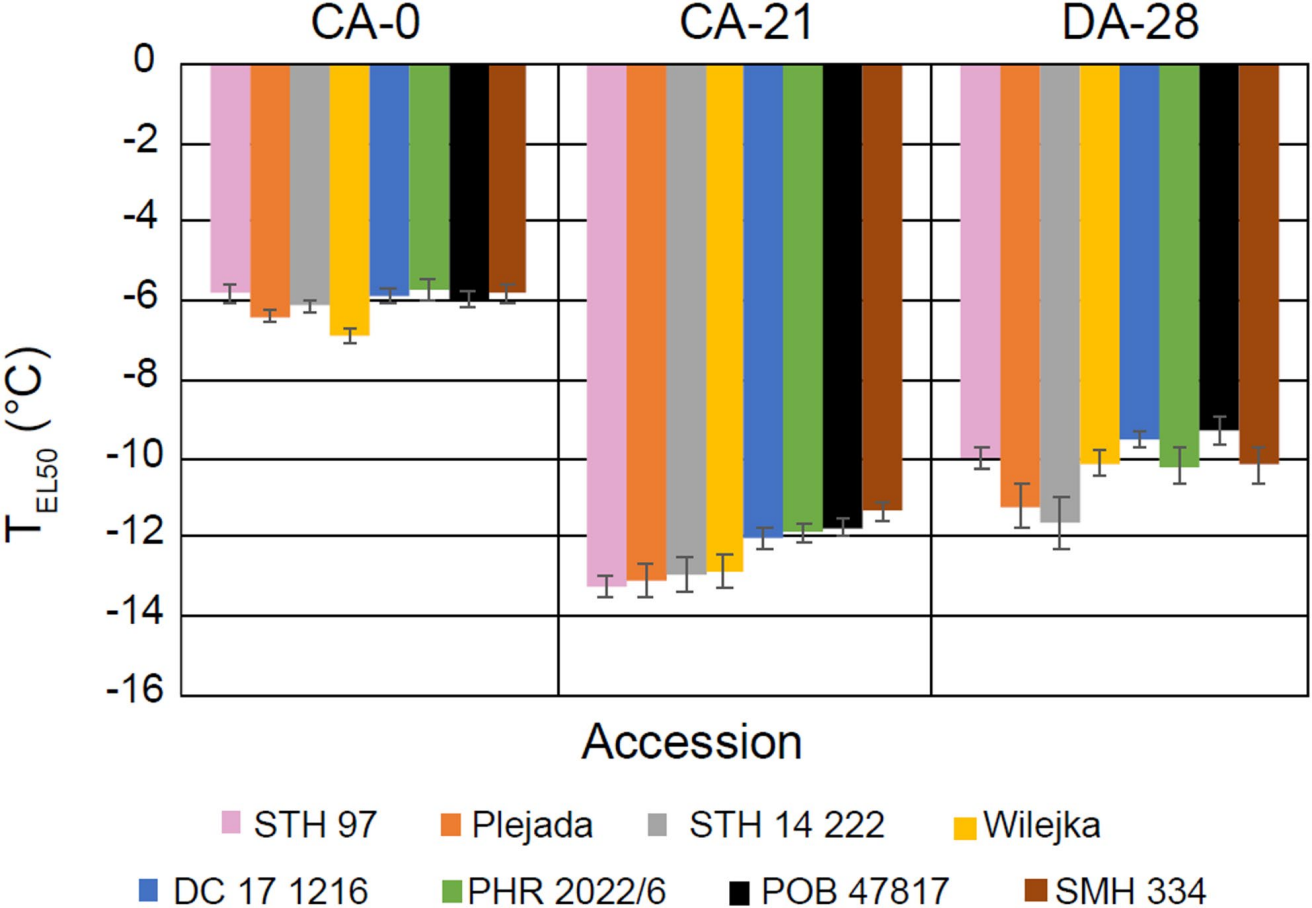
Freezing tolerance of selected wheat lines and varieties determined as the freezing temperature causing 50% electrolyte leakage from leaves (TEL50). The values are presented for three time points: non-acclimated plants (CA-0), after cold acclimation (CA-21), and after 7-day de-acclimation (DA-28). Vertical error bars represent 95% confidence intervals (α = 0.05) calculated for the LT50 estimates using the normal (Wald) approximation. Significant differences between means at *P* ≤ 0.05 were inferred from non-overlapping confidence intervals

Measurements of the cell membrane integrity showed that the freezing tolerance ranking of the tested varieties and lines differed between cold acclimated, non-acclimated and de-acclimated plants (Fig. 1). In the non-acclimated state (CA-0), the most freezing-tolerant varieties were Wilejka and Plejada, which also belonged to the group of the most freezing-tolerant varieties after cold acclimation. The freezing tolerance of the remaining six lines was similar in CA-0. After de-acclimation, the most freezing-tolerant accessions were STH 14,222 and Plejada. The freezing tolerance of the others was similar, with the exception of the significantly weaker POB 47,817 and DC 17 1216 lines (Fig. 1).

In addition, the extent to which chlorophyll fluorescence measurements (after freezing of cut leaves of cold acclimated and de-acclimated plants) can be used to determine the response of plants to de-acclimation was examined (Tables S2 and S3).

**Fig. 2:**
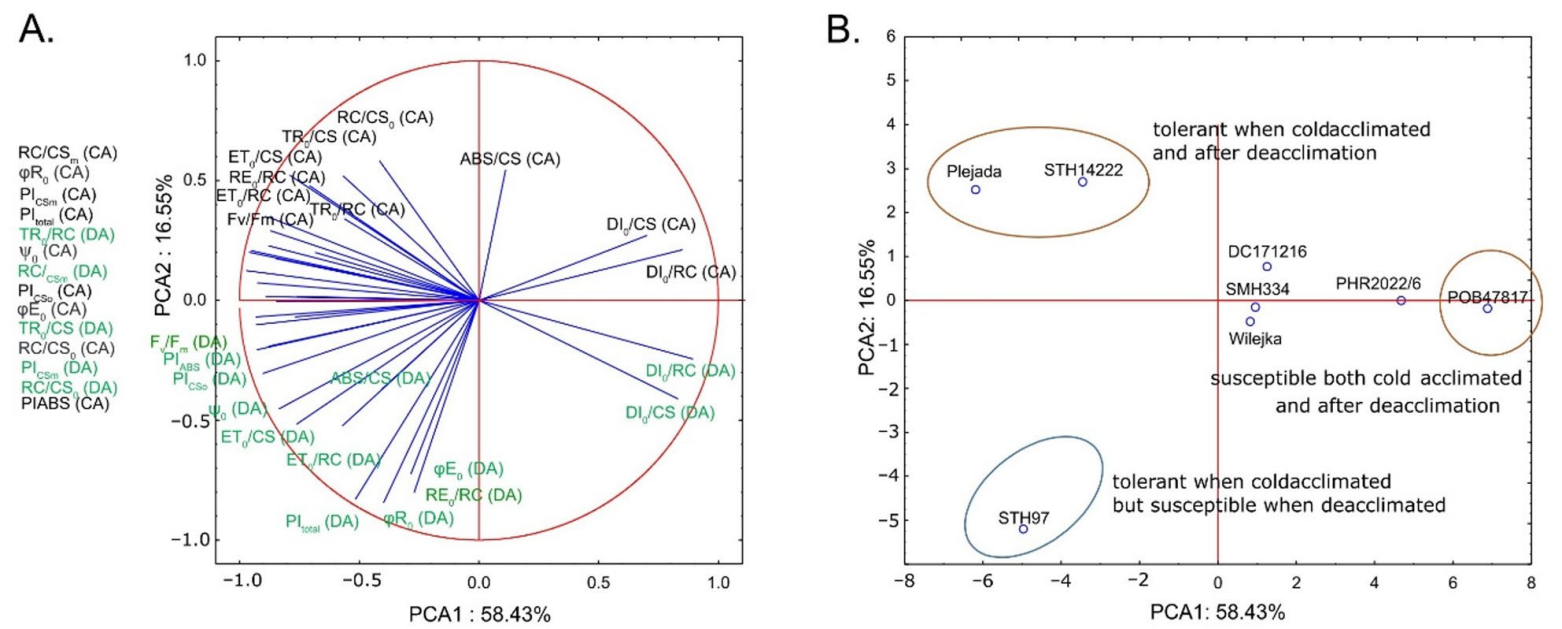
Results of the principal component analysis for chlorophyll fluorescence measurements performed for the eight selected wheat lines and varieties after cold acclimation (CA) and de-acclimation (DA, green color in panel **A**). Panel **B** shows the differentiation of the tested varieties and lines based on the measurement results. In panel **A**, chlorophyll fluorescence parameters listed in the left margin describe not tagged vectors in the left part of the graph

Principal component analysis (PCA) separated cold acclimated (CA) and de-acclimated (DA) plants based on chlorophyll fluorescence parameters (Fig. 2). The parameters analysing the results of chlorophyll fluorescence measurements taken in hardened and unhardened plants differentiated the pool of studied accessions (Fig. 2A). This applies to virtually all parameters, with a few exceptions, e.g. RC/CS_0_. It may suggest the different genetical background of freezing tolerance of photosynthetic apparatus in cold-acclimated and de-acclimated plants. The DI parameters (DI_0_/CS, DI_0_/RC) showed a distinct clustering because energy dissipation in the form of heat increases as damage to PSII progresses in contrary to other parameters.

The PCA grouped the tested accessions depending on the reaction of their photosynthetic apparatus to freezing after cold acclimation and de-acclimation (Fig. 2B). The upper left quadrant of Fig. 2B contains Plejada and STH 14,222, which, according to cell membrane integrity measurements, are classified as freezing-tolerant both after cold acclimation and de-acclimation. In the lower left quadrant is STH 97, which, despite its high freezing tolerance in the cold-acclimated state, is the least freezing-tolerant of the tested group after de-acclimation. On the contrary, the right half of the graph shows POB 47,817, which has the lowest freezing tolerance both after hardening and de-acclimation. Despite its high freezing tolerance shown in both survival rate and TEL50, Wilejka was grouped together with two susceptible lines: DC 17 1216 and SMH 334. This suggests a weaker correlation of survival rate after freezing and the level of damage to the photosynthetic apparatus in this cultivar.

### Susceptible accessions show no freezing survival after de-acclimation

Tolerant accessions presented very high survival rate (73–93%) after freezing in de-acclimated state. All sensitive lines displayed survival rate of 0% as well as low values of selected chlorophyll fluorescence parameters. However, some of the susceptible accessions showed no apparent damage to the photosynthetic apparatus (Table 1). This shows that although chlorophyll fluorescence can usually be used as an indicator of freezing tolerance, it is not always highly correlated with the actual plant survival.

**Table 1:** Summary of breeding lines and cultivars identified as most tolerant and most sensitive to active de-acclimation, together with the results obtained. S - sensitive, T - tolerant, FR-T - survival rate [%] after freezing in de-acclimated state, PI _CSm_- performance index in excited PSII in relation to the photosynthesising surface area of the sample (CS) after freezing in de-acclimated state, RC/CS_m_ - density of reaction centres capable of reducing plastoquinone (QA) at time t_max_ after freezing in de-acclimated state. The values of OJIP parameters should be interpreted as relative, unitless parameters

Accession	S/ T	FR-T	PI_CSm_	RC/CS_m_
STH97	T	73%	228	247
STH 14,222	T	93%	451	298
Plejada	T	87%	463	218
Wilejka	T	85%	192	493
DC 17 1216	S	0%	671	336
POB 47,817	S	0%	247	265
PHR 2022/6	S	0%	55	143
SMH 334	S	0%	58	159

### Active de-acclimation results predominantly in a downregulation of genes expressed in cold-acclimated state

To analyse cold acclimation and de-acclimation processes at the molecular level, we performed transcriptomic analysis by RNA-seq on the leaves of selected de-acclimation tolerant and susceptible wheat accessions. In wheat accessions tolerant to de-acclimation, 4960 genes (2385 with upregulated and 2575 with downregulated expression) were differentially expressed between the cold acclimated state (CA-21) and the non-acclimated state (CA-0) at *P* ≤ 0.01. Among accessions susceptible to de-acclimation, 2479 transcripts (612 with upregulated and 1867 with downregulated expression) were identified between CA-21 and CA-0 at *p* ≤ 0.01 (Fig. S1).

**Fig. 3:**
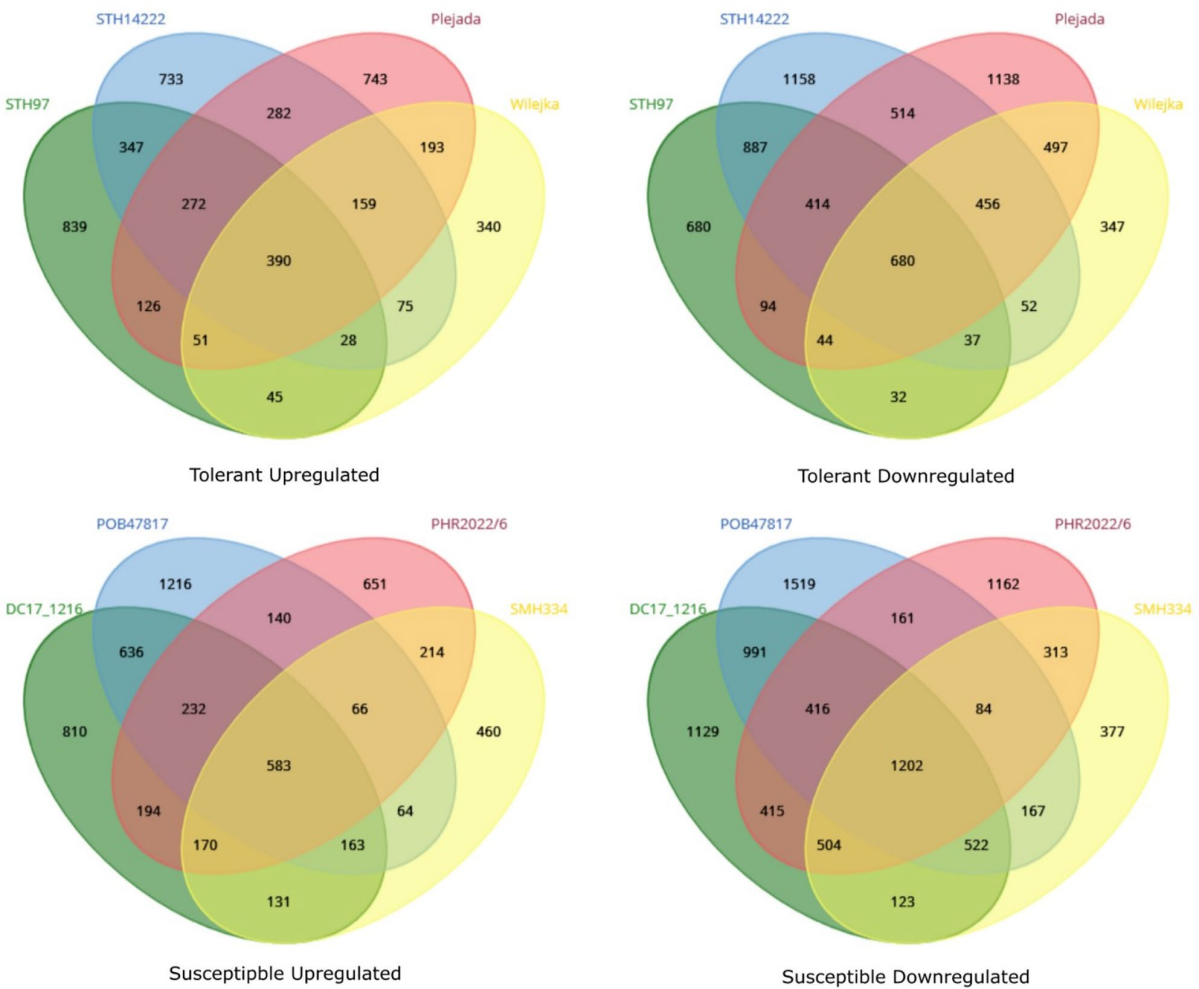
Differentially expressed genes (DEGs) identified between cold-acclimated (CA-21) and de-acclimated (DA-28) wheat accessions. DEGs were defined as genes showing |log₂FC| ≥ 1 and *P* ≤ 0.01. The analysis was performed separately for accessions classified as tolerant or susceptible to active de-acclimation. Upregulated genes represent transcripts with higher expression in DA-28 compared to CA-21, whereas downregulated genes represent transcripts with lower expression in DA-28 relative to CA-21. Numbers indicate the total counts of DEGs in each category

Between the de-acclimated state (DA-28) and the cold acclimated state (CA-21), 1070 genes (390 with upregulated and 680 with downregulated expression) were differentially expressed at *p* ≤ 0.01 in the wheat accessions tolerant to de-acclimation. Among the accessions susceptible to de-acclimation, 1785 (583 with increased and 1202 with downregulated expression) were identified between DA-28 and CA-21 at *p* ≤ 0.01 (Fig. 3).

Also, in the wheat accessions tolerant to de-acclimation, 3762 genes (1570 with upregulated and 2192 with downregulated expression) were differentially expressed between the de-acclimated state (DA-28) and the non-acclimated state (CA-0, control) at *p* ≤ 0.01. Among the susceptible to de-acclimation accessions, 3075 genes (1223 with upregulated and 1852 with downregulated expression) were identified between DA-28 and CA-0 at *p* ≤ 0.01 (Fig. S2).

**Fig. 4:**
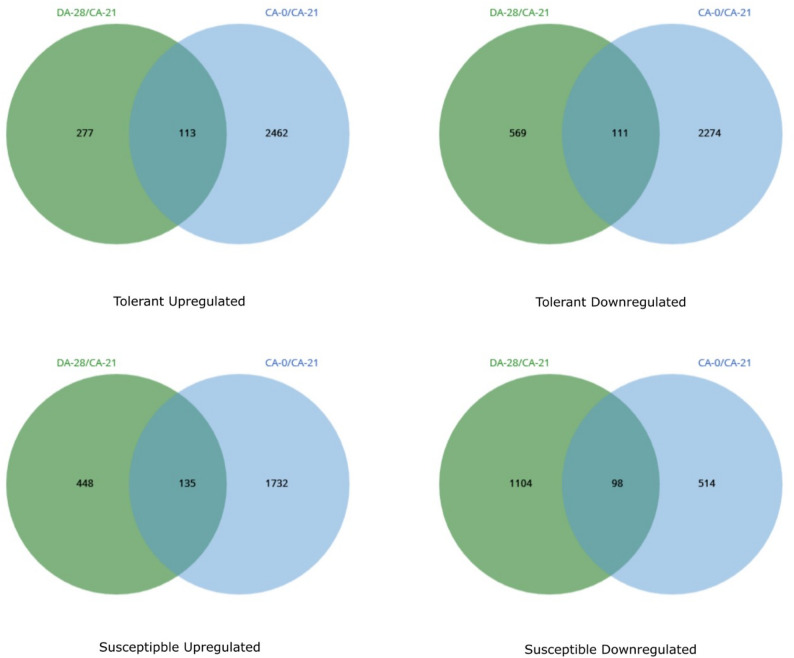
Differentially expressed genes (DEGs) specific to the active de-acclimation process in wheat accessions differing in tolerance. DEGs were defined as genes showing |log₂FC| ≥ 1 and *P* ≤ 0.01 and were identified by comparing gene sets from CA-21 vs. CA-0 (cold acclimation) and DA-28 vs. CA-21 (de-acclimation), allowing the identification of genes uniquely associated with de-acclimation. Results are shown separately for tolerant and susceptible accessions. Upregulated and downregulated refer to changes in expression in DA-28 relative to CA-21. Numbers indicate the size of each DEG group

As a result of the comparison of common differentially expressed genes (DEGs) from the CA-0/CA-21 and DA-28/CA-21 processes, information was obtained on DEGs specific only to active de-acclimation, which are not just the reverse of the cold acclimation process. 846 DEGs (277 with an upregulation in expression and 569 with a downregulation) were specific only for conditions imitating warm spell during the mid-winter period in de-acclimation-tolerant wheat plants (*p* ≤ 0.01) (Fig. 4). Furthermore, 1552 genes (448 with upregulated expression and 1104 with downregulated expression) were specifically expressed only after de-acclimation in sensitive wheat accessions (*p* ≤ 0.01) (Fig. 4).

**Fig. 5:**
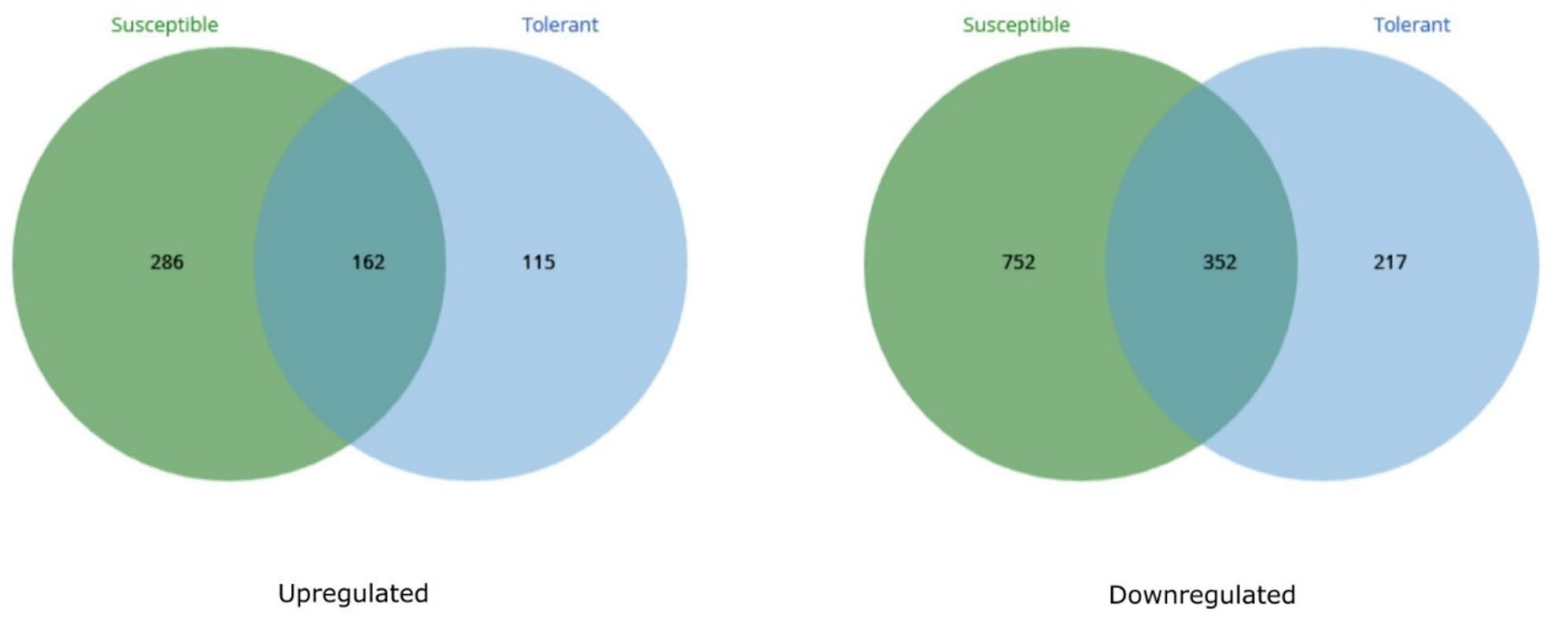
Overlap of differentially expressed genes (DEGs) specific to the active de-acclimation process in tolerant and susceptible wheat accessions. DEGs were defined as genes showing |log₂FC| ≥ 1 and *P* ≤ 0.01. The diagram illustrates the number of de-acclimation-specific DEGs shared between tolerant and susceptible accessions, as well as those unique to each group. Upregulated and downregulated refer to expression changes in DA-28 relative to CA-21

Only 514 DEGs (162 with upregulated and 352 with downregulated expression, *p* ≤ 0.01) specific only to de-acclimation were common to both de-acclimation-tolerant and de-acclimation-susceptible winter wheat accessions (Fig. 5). The number of DEGs characteristic of the group of de-acclimation-sensitive winter wheat accessions significantly exceeded the number of both commonly expressed and tolerant-specific differentially expressed genes (Fig. 5).

**Fig. 6:**
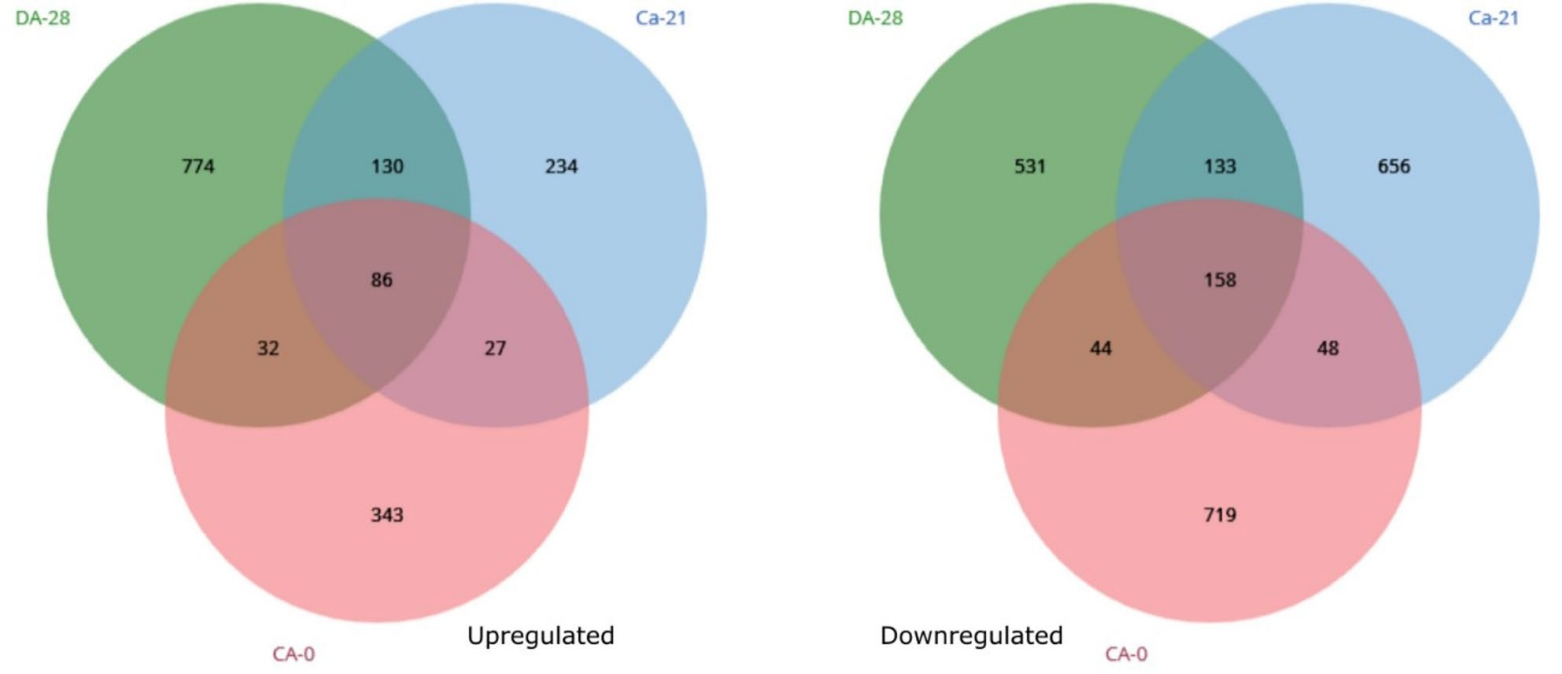
Differentially expressed genes (DEGs) between wheat accessions tolerant and susceptible to active de-acclimation at three time points. DEGs were defined as genes showing |log₂FC| ≥ 1 and *P* ≤ 0.01. Comparisons were performed between tolerant and susceptible accessions at CA-0 (before cold acclimation), CA-21 (after cold acclimation), and DA-28 (after de-acclimation). Upregulated genes represent transcripts with higher expression in tolerant accessions relative to susceptible ones, whereas downregulated genes represent transcripts with lower expression in tolerant accessions

Another approach to determine the molecular mechanism of tolerance to active de-acclimation in wheat was to compare the common genes expressed in tolerant accessions with the common DEGs in the group of susceptible lines at each of the three measurement points. So these were not genes related to the de-acclimation process, but all genes expressed at a given time (CA-0, CA-21 or DA-28). The DEG lists obtained for each measurement point for the tolerant accessions against the sensitive ones were compared with each other (Fig. 6).

Among the differentially expressed genes in tolerant accessions relative to sensitive accessions, 1,305 (774 with upregulated and 531 with downregulated expression relative to sensitive lines, *p* ≤ 0.01) were observed as specific to the measurement point after seven days of de-acclimation (DA-28) (Fig. 6).

Since transcripts from the DA-28 pool (Fig. 6, green areas) refer to all genes undergoing expression at the DA-28 measurement point (i.e. after 7 days of de-acclimation), and transcripts from the DA-28/CA-21 pool (Fig. 4, green areas) refer only to genes that were differentially expressed between the DA-28 and CA-21 time points (i.e. the difference between the de-acclimated plants and the cold acclimated plants), it was decided to compare the two pools to isolate transcripts associated with the DA-28 time point, but not resulting from de-acclimation. This comparison may provide additional information on the differences between the tolerant and the sensitive accessions studied in this project. A comparison of DEGs specific to measurement point DA-28 (tolerant versus sensitive accessions, Fig. 6, green areas) and DEGs specific to the de-acclimation process in all accessions (Fig. 4, green areas) revealed only 189 common genes. There were 1116 DEGs associated with the DA-28 measurement point, but not with the DA-28/CA-21 change (Fig. S3).

### Active de-acclimation triggers changes in the expression of phytohormone-related genes in wheat

**Fig. 7:**
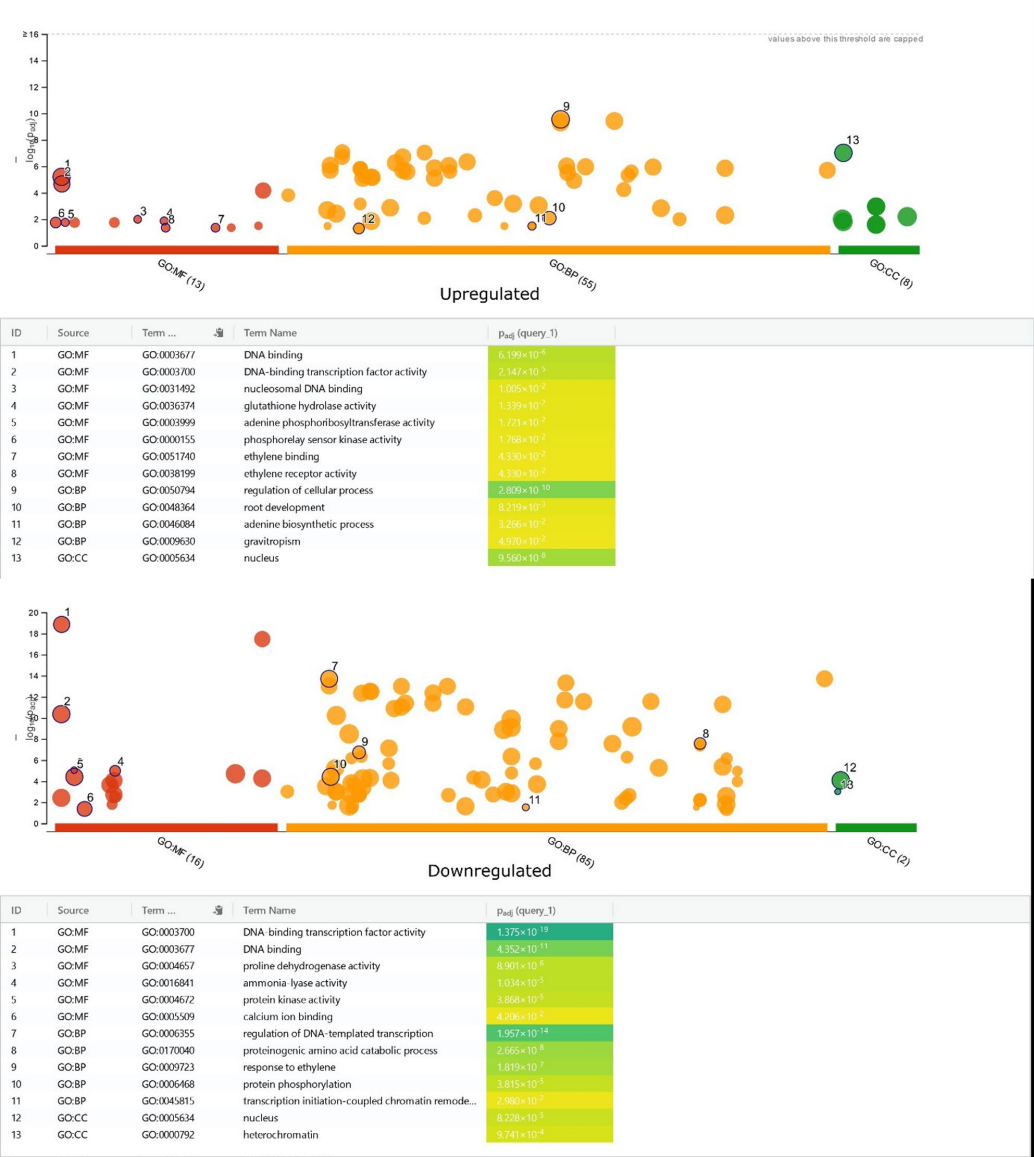
Gene ontology (GO) enrichment analysis for differentially expressed genes (DEGs) specific to the active de-acclimation process and common to tolerant and susceptible wheat accessions. DEGs (|log₂FC| ≥ 1, FDR < 0.05) were identified based on DA-28 vs. CA-21 comparison. The upper panel shows GO terms enriched among genes upregulated in DA-28, while the lower panel shows GO terms enriched among downregulated genes. Categories are grouped into Biological Process (BP), Molecular Function (MF), and Cellular Component (CC). Circle size corresponds to the number of genes associated with a given GO term

Among the DEGs specific to the de-acclimation process and common to both tolerant and susceptible accessions, we identified a subset of genes showing upregulated expression relative to the cold-acclimated state. Within this group, transcripts related to root development (including ‘root development’ and ‘gravitropism’) were overrepresented. In addition, genes associated with ethylene perception (including ‘ethylene binding’ and ‘ethylene receptor activity’) were also significantly enriched (Fig. 10). Ethylene perception-related transcripts were also over-represented among DEGs with downregulated expression relative to CA-21 (‘response to ethylene’). Another prominent function in this group of DEGs was proline dehydrogenase activity (Fig. 7).

**Fig. 8:**
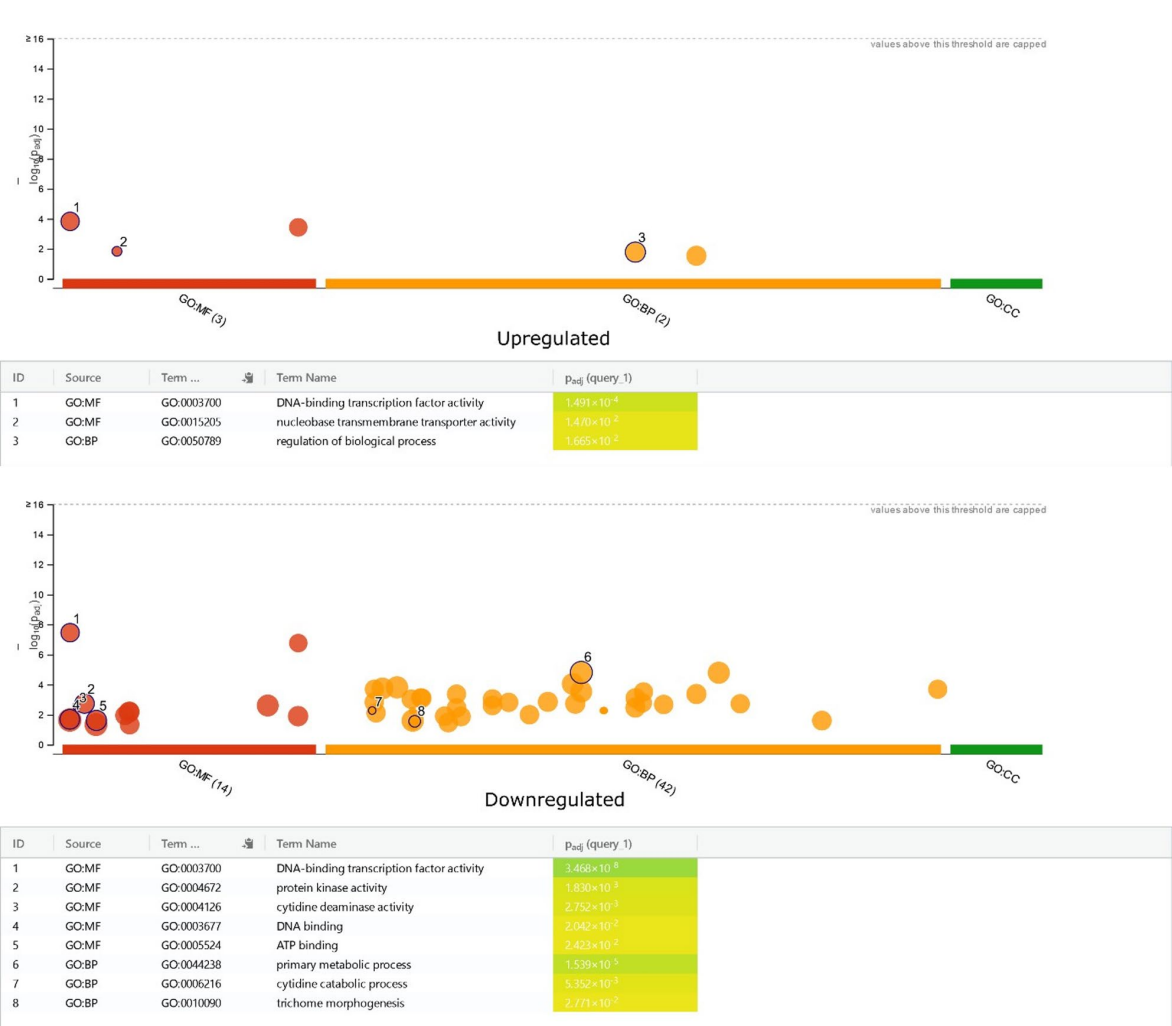
Gene ontology (GO) enrichment analysis for differentially expressed genes (DEGs) specific to the active de-acclimation process in tolerant wheat accessions. DEGs (|log₂FC| ≥ 1, FDR < 0.05) were identified based on DA-28 vs. CA-21 comparison. The upper panel shows GO terms enriched among genes upregulated in DA-28, while the lower panel shows GO terms enriched among downregulated genes. Categories are grouped into Biological Process (BP), Molecular Function (MF), and Cellular Component (CC). Circle size corresponds to the number of genes associated with each GO term

Among the DEGs characteristic only of the de-acclimation process in tolerant accessions, no overrepresentation of any processes, functions, or cellular elements was observed to allow inference of active de-acclimation tolerance mechanisms in wheat, except for the down-regulated expression of biological processes related to trichome morphogenesis compared to the CA-21 time point (Fig. 8).

**Fig. 9:**
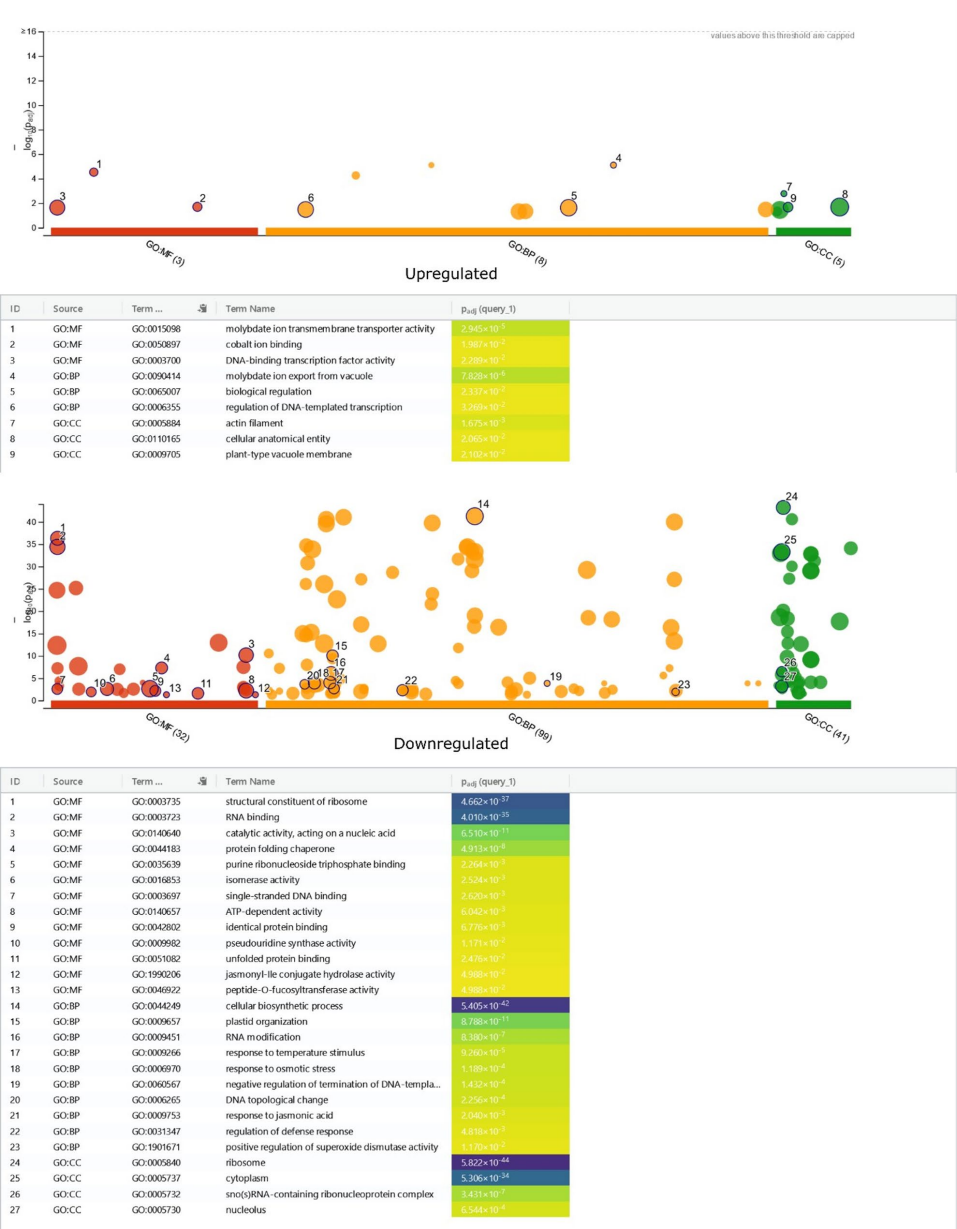
Gene ontology (GO) enrichment analysis for differentially expressed genes (DEGs) specific to the active de-acclimation process in susceptible wheat accessions. DEGs (|log₂FC| ≥ 1, FDR < 0.05) were identified based on DA-28 vs. CA-21 comparison. The upper panel shows GO terms enriched among genes upregulated in DA-28, while the lower panel shows GO terms enriched among downregulated genes. Categories are grouped into Biological Process (BP), Molecular Function (MF), and Cellular Component (CC). Circle size corresponds to the number of genes associated with each GO term

Among the DEGs specific only to the de-acclimation process, an overrepresentation of transcripts related to metal ion transport (“molybdate ion transmembrane transporter activity”, “cobalt ion binding”, “molybdate ion export from vacuole” and a related site in the cell: “plant-type vacuole membrane” were observed in sensitive accessions, with upregulated expression compared to the de-acclimated state) (Fig. 9).

Differentially expressed genes (DEGs) exhibiting reduced expression in DA-28 relative to CA-21 within the group of susceptible accessions were considerably more abundant than those displaying upregulated expression. Consequently, the Gene Ontology (GO) analysis revealed a substantially larger number and greater diversity of enriched functional categories. Among them, transcripts related to osmotic and temperature stress response (“response to osmotic stress”, “response to temperature stimulus”), transcripts related to jasmonic acid perception (“response to jasmonic acid”), as well as transcripts related to plant defence response (thus also stress response: “regulation of defence response”, “positive regulation of superoxide dismutase activity”) provide more detailed information about the mechanisms of sensitivity (Fig. 9).

**Fig. 10:**
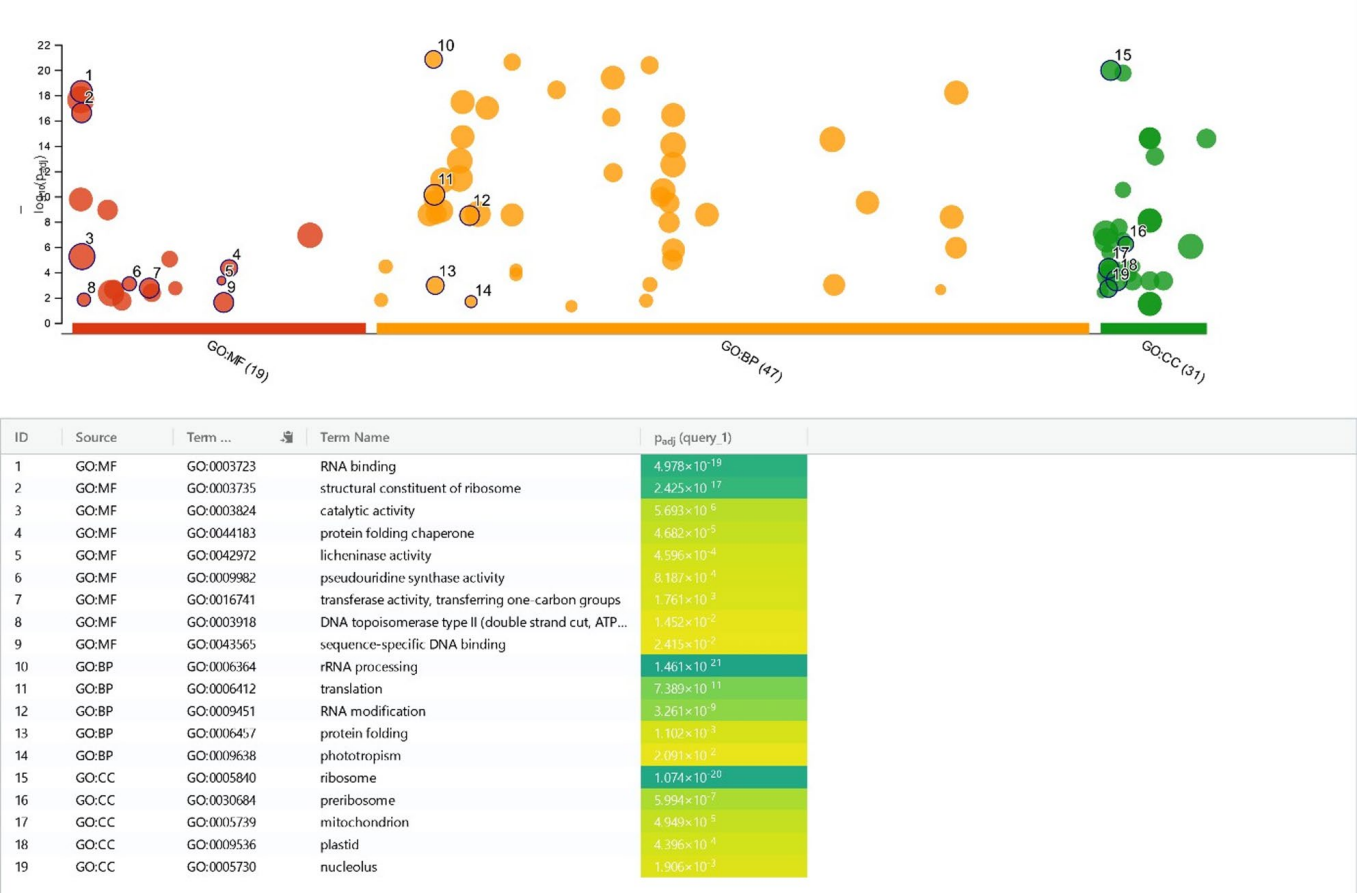
Gene ontology (GO) enrichment analysis for differentially expressed genes (DEGs) distinguishing tolerant and susceptible wheat accessions at the de-acclimated stage (DA-28). DEGs (|log₂FC| ≥ 1, FDR < 0.05) represent genes differentially expressed between tolerant and susceptible accessions at DA-28, excluding those directly associated with the de-acclimation transition (DA-28 vs. CA-21). Categories are grouped into Biological Process (BP), Molecular Function (MF), and Cellular Component (CC). Circle size corresponds to the number of genes associated with each GO term

Among the DEGs specific to the DA-28 time point (but not to the de-acclimation process understood as DA-28 versus CA-21) in tolerant versus sensitive accessions, in addition to an overrepresentation of very general processes, functions and locations in the cell, an overrepresentation of transcripts related to phototropism and endo-1,3(4)-beta-glucanase (“licheninase activity”) was observed (Fig. 10).

**Fig. 11:**
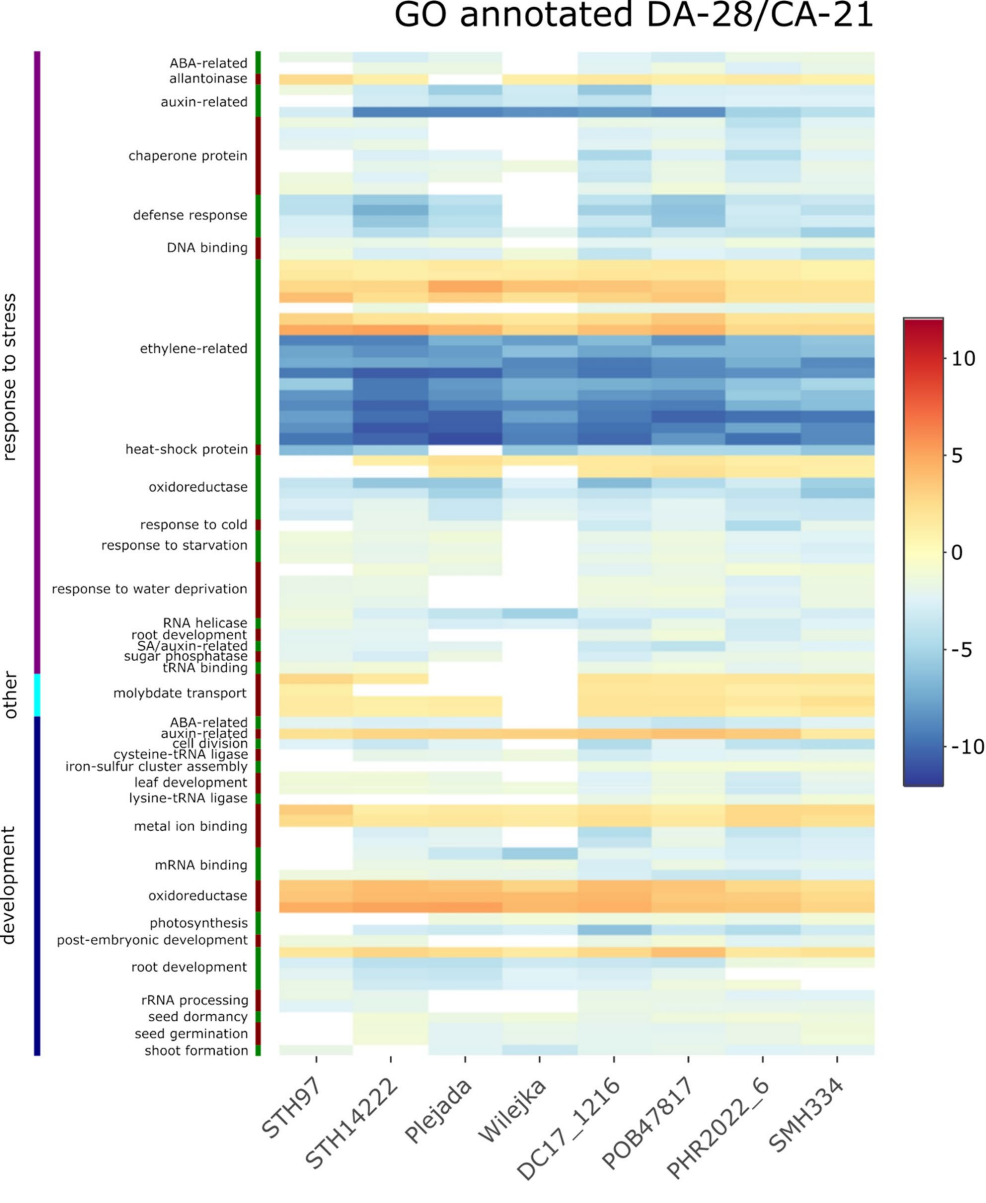
Heatmap of gene expression changes associated with the active de-acclimation process in wheat accessions. The heatmap shows log₂ fold change (DA-28/CA-21) for selected genes across eight accessions. Rows represent genes, and columns represent accessions. The first four columns correspond to accessions tolerant to de-acclimation, and the last four correspond to susceptible accessions. Colour scale indicates the magnitude and direction of expression changes

**Fig. 12:**
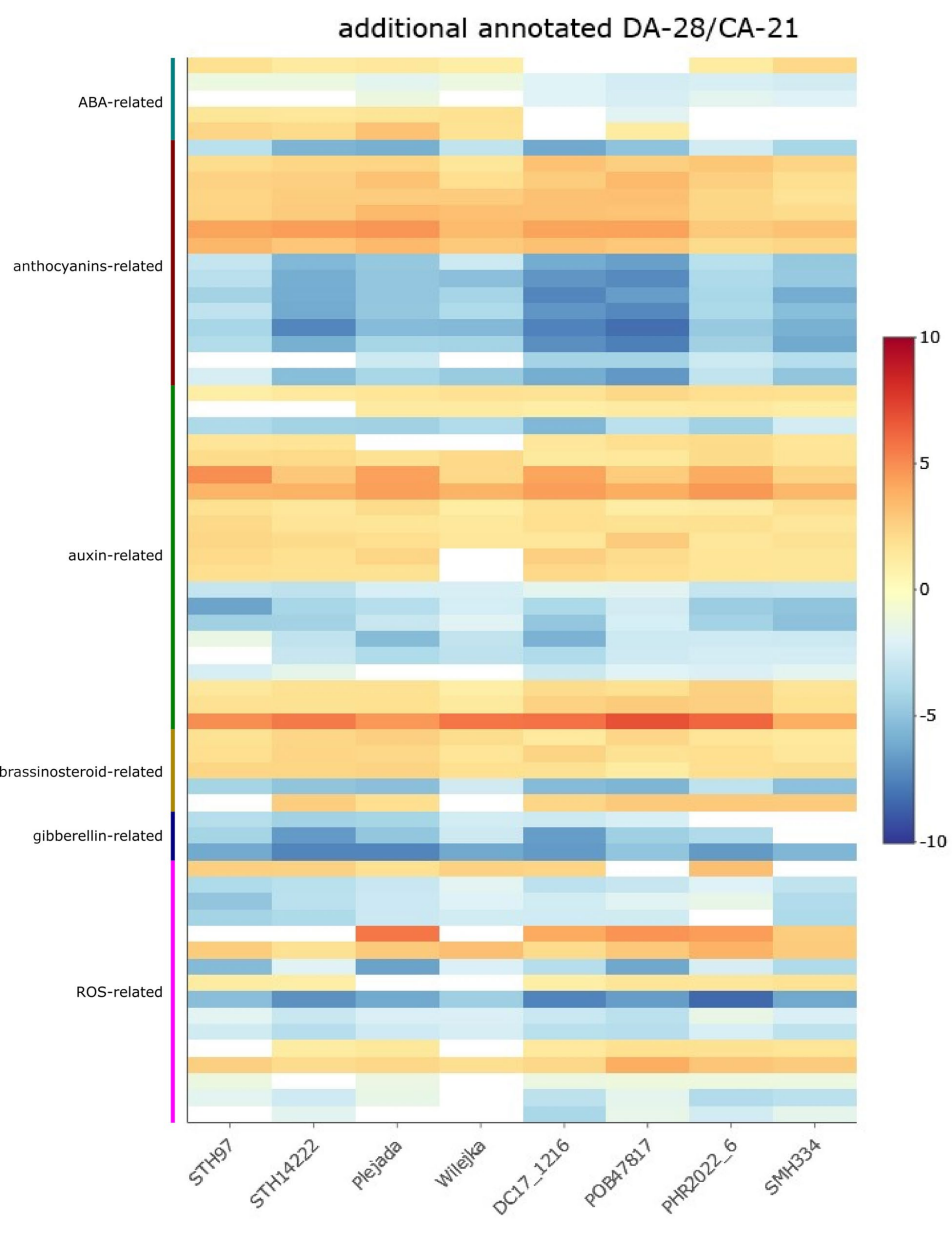
Heatmap of expression changes for selected stress-related genes during de-acclimation in wheat accessions. The heatmap shows log₂ fold change (DA-28/CA-21) for selected genes across eight accessions. Rows represent genes, and columns represent accessions. The first four columns correspond to tolerant accessions, and the last four to susceptible accessions. Colour scale indicates relative expression changes

For genes present in overrepresented processes and functions related to stress response and development, an analysis of expression changes due to de-acclimation (DA-28/CA-21) in each accession was performed (Fig. 11). An analogous analysis was additionally carried out for selected other stress response-related genes that did not exceed the significance threshold in the gene ontology analysis (Fig. 12), and for selected genes undergoing differential expression in tolerant versus sensitive accessions at the DA-28 time point (combined genes from the ontology analysis and additional genes selected by function and repetition criterion, Fig. S4).

Analysis of the expression of selected genes in all the test accessions revealed that most of the DEGs identified as characteristic of one of the groups (tolerant or sensitive to de-acclimation) also underwent differential expression in part (sometimes ¾) of the other group (Fig. 11). This makes it difficult to identify the mechanisms involved in tolerance to de-acclimation and shows that most of the observed expression changes are probably universal to the response to active de-acclimation in wheat. Distinctively large expression changes were observed in all accessions in the ethylene perception-related gene group. There was both an increase and a decrease in expression (Fig. 11).

Among the other selected stress response-related genes differentially expressed between the DA-28 and CA-21 time points, most were plant hormone-related genes. In addition, genes related to the response to reactive oxygen species (“ROS-related”) and genes related to anthocyanin synthesis and metabolism (“anthocyanins-related”) also formed distinctive groups (Fig. 12). Among these additional DEGs, the majority are also transcripts differentially expressed in both tolerant and sensitive wheat subjects. However, in some cases, the differences in expression between these groups are quite evident, and especially in the case of the “abscisic acid 8’-hydroxylase 1-like” (abscisic acid 8’-hydroxylase 1-like) gene belonging to the “ABA-related” group. In the susceptible group, no change in the expression of this gene was observed in ¾ between DA-28 and CA-21, and where it was observed (POB47817), the direction of the change is the opposite of that in the group of tolerant accessions (Fig. 12).

When analysing the expression of genes specific to the DA-28 time point, but not to the de-acclimation process understood as a DA-28/CA-21 change, normalized gene expression values of TPM (Transcript per Million), which relates the number of reads of a given sequence to the length of the gene (RPK - reads per kilobase), and then to all RPKs in a given sample divided by one million, were used. Using log_2_FC expression change values between the two time points was not possible because, for this comparison, log_2_FC referred to all tolerant accessions relative to all sensitive accessions. The expression analysis thus performed in all accessions confirmed the potential role of genes encoding licheninase and the high expression levels of genes related to ethylene perception (Figure S4).

### qPCR provides additional arguments for the role of ABA hydroxylase and gibberellin oxidase genes in the mechanism of tolerance to active de-acclimation in wheat

Based on the expression results of a given gene in each accession and its adherence to one of the overrepresented functional groups, 7 candidate genes were selected for verification by RT-qPCR (Table S5). In addition, one gene associated with the ethylene biosynthesis pathway was selected due to the high overrepresentation of ethylene-related genes in the RNAseq results. The *Triticum aestivum TraesCS4A02G111500* gene (*ETO1-like*), which is related to the *ETHYLENE OVERPRODUCTION 1 (ETO1)* gene identified in *Arabidopsis thaliana*, was selected for the analysis [19–22].

**Fig. 13:**
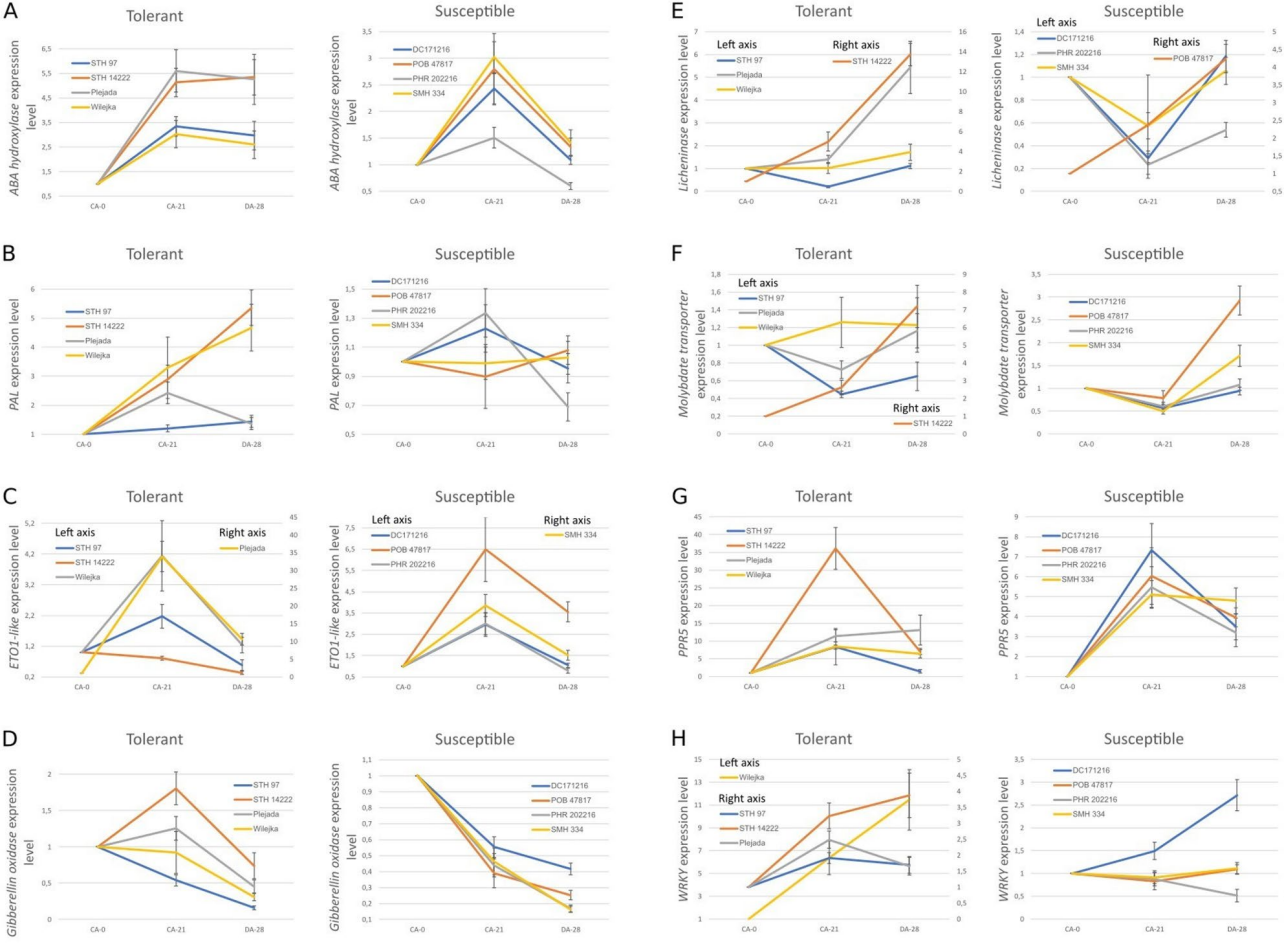
Relative expression profiles of selected genes in wheat accessions differing in tolerance to active de-acclimation. Gene expression was measured by RT-qPCR at three time points: CA-0 (before cold acclimation), CA-21 (after 21 days of cold acclimation), and DA-28 (after 7 days of de-acclimation). Expression levels are shown separately for tolerant (left panels) and susceptible (right panels) accessions and are normalized relative to CA-0. Error bars represent standard errors based on biological replicates. Panels: (**A**) *ABA hydroxylase*, (**B**) PAL, (**C**) Gibberellin oxidase, (**D**) ETO1-like, (**E**) Licheninase, (**F**) Molybdate transporter, (**G**) PPR5, (**H**) WRKY

Most of the tested candidate genes showed similar expression profiles to those noted in the RNA-seq experiment. Additionally, most of the observed divergences were related rather to the magnitude of the change in expression between two given timepoints, than to direction of this change (up- or downregulation) (Table S5). With a few exceptions all of the studied genes, both the 7 chosen for verification of RNA-seq, and the ethylene biosynthesis-related *ETO1*-like gene showed changes in expression caused by cold acclimation and de-acclimation (Fig. 13).

Two of the tested genes, namely *ABA hydroxylase*, and *Gibberellin oxidase*, showed visible differences in their expression profiles between the tolerant and the susceptible accession group. These differences were most pronounced in the case of *ABA hydroxylase* gene. Its expression was upregulated between the CA-0 and CA-21 timepoints, but in the susceptible accessions, it was downregulated after de-acclimation to more or less the level from before cold-acclimation. In tolerant accessions, the level of expression after de-acclimation stayed similar to the level after cold-acclimation (Fig. 13A).

In the case of *Gibberellin oxidase* gene, most of the tolerant accessions showed an upregulation or no change in expression caused by cold acclimation, followed by a strong downregulation after de-acclimation treatment. On the other hand, all of the susceptible accessions displayed a significant downregulation of this gene between CA-0 and CA-21 timepoints, and further downregulation after de-acclimation (Fig. 13D).

**Fig. 14:**
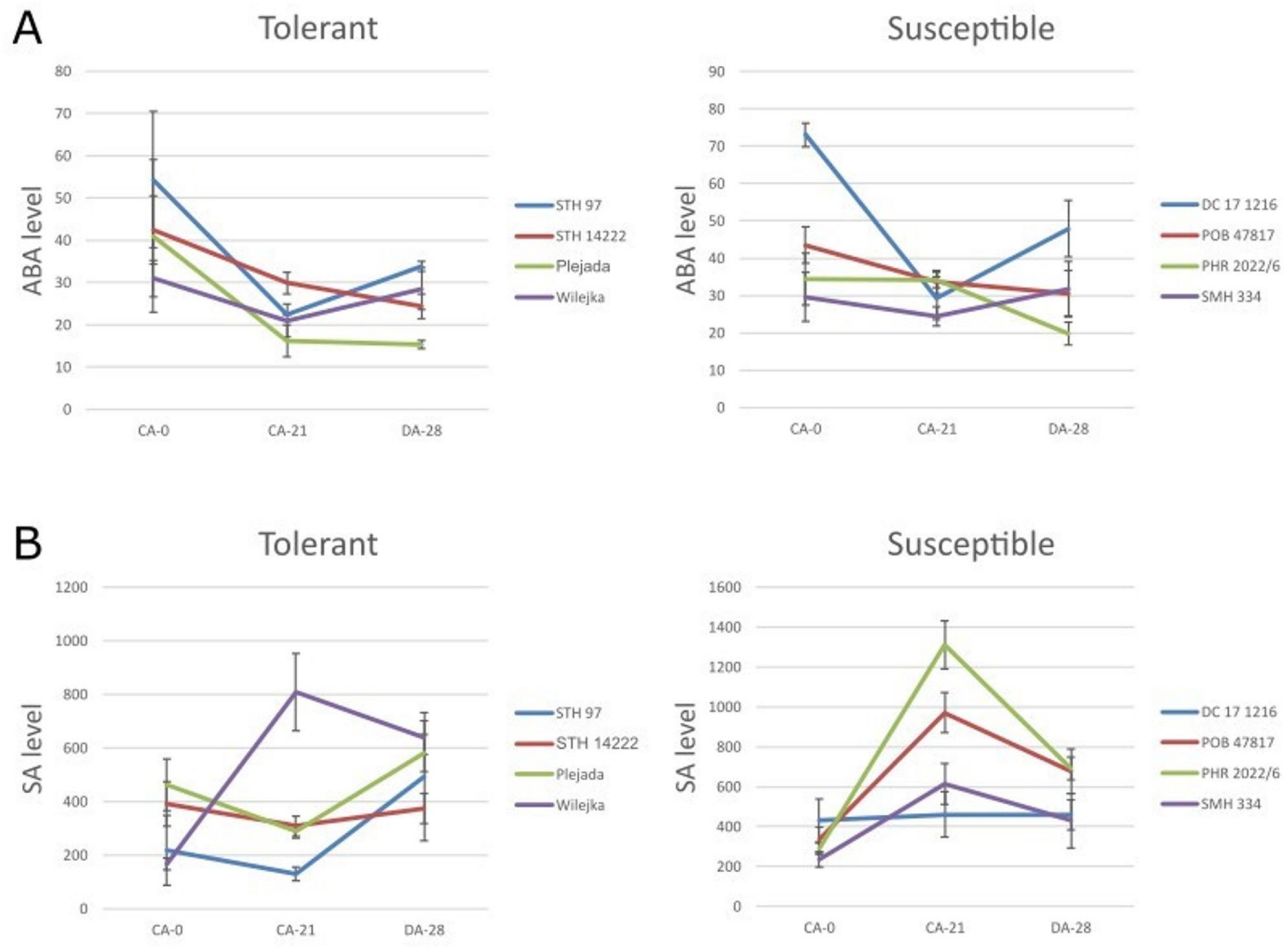
Changes in levels of acidic phytohormones in wheat accessions differing in tolerance to active de-acclimation. Hormone levels were measured at three time points: CA-0 (before cold acclimation), CA-21 (after cold acclimation), and DA-28 (after de-acclimation). Results are shown separately for tolerant (left panels) and susceptible (right panels) accessions. Panels: (**A**) abscisic acid (ABA), (**B**) salicylic acid (SA). Error bars represent standard errors of biological replicates

**Fig. 15:**
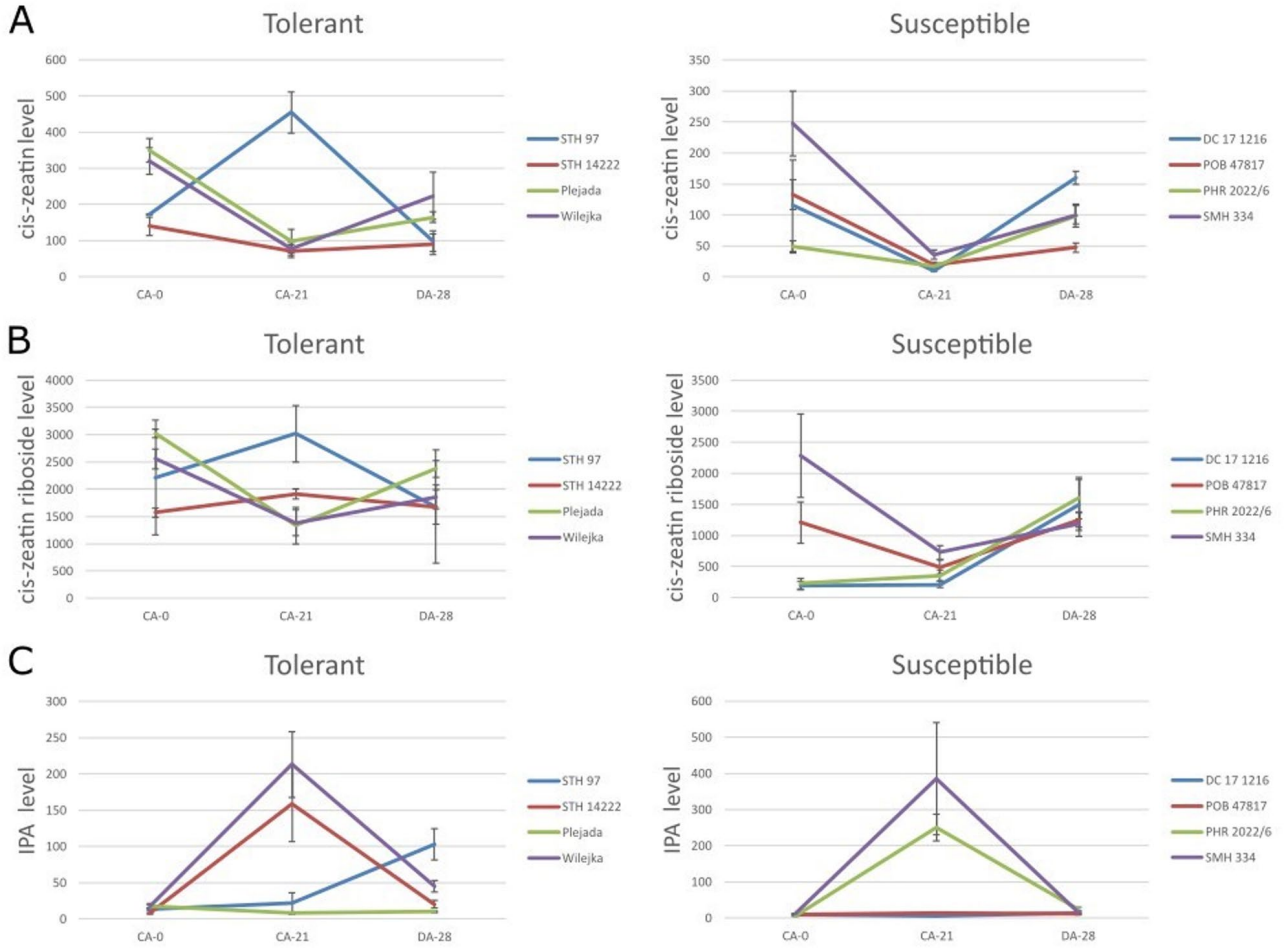
Changes in cytokinin levels in wheat accessions differing in tolerance to active de-acclimation. Cytokinin species were quantified at three time points: CA-0, CA-21, and DA-28. Results are shown separately for tolerant (left panels) and susceptible (right panels) accessions. Panels: (**a**) cis-zeatin, (**b**) cis-zeatin riboside, (**c**) isopentenyladenine (iPA). Error bars represent standard errors of biological replicates

Of all tested plant hormones, only ABA, SA, and selected cytokinin species showed results that could be connected to differences in de-acclimation tolerance between studied accessions (Figs. 14 and 15). The level of ABA decreased after 3-week period of acclimation to cold in all of the tolerant accessions and in most of the susceptible ones. However, only in one susceptible accession, namely DC 17 1216, this decrease was as dramatic, as in the tolerant accessions. The rest of the susceptible accessions showed a mild decrease or lack of change between the CA-0 and CA-21 timepoints. The ABA level change caused by de-acclimation showed no common pattern for any of the accession groups (Fig. 14A).

SA was the only tested plant hormone which showed visible difference in its level fluctuation pattern between the tolerant and the susceptible group of accessions. In three out of four tolerant accessions, the SA level decreased after 3-week cold acclimation period. SA level increased after one week of de-acclimation to a level higher than recorded in the CA-0 timepoint. At the same time, most susceptible accessions (also ¾) showed a completely opposite pattern: an increase due to cold acclimation, and a decrease caused by de-acclimation (Fig. 14B).

In the case of cytokinin species, the susceptible accessions shared a common pattern of cis-zeatin and cis-zeatin riboside level changes, while the tolerant group was less consistent. The level of cis-zeatin riboside of Plejada and Wilejka showed the same pattern of changes throughout the experiment as in the susceptible accessions, while STH 97 and STH 14,222 shared a reverse trend. The level of IPA increased after cold acclimation and then decreased after de-acclimation to the level recorded in CA-0 in one half of the accessions, irrespective of their tolerance (Fig. 15).

## Discussion

### De-acclimation as a distinct physiological and stress-related process

The present study demonstrates that active de-acclimation in winter wheat represents a distinct physiological process rather than a simple reversal of cold acclimation. A substantial proportion of differentially expressed genes was specific either to cold acclimation or to de-acclimation, with only limited overlap between the two processes. This indicates that the molecular response to warming is qualitatively different from the response to cold. This observation is consistent with previous studies in cereals, where de-acclimation has been shown to involve specific regulatory pathways rather than a passive loss of cold-induced traits [1, 6].

Similar conclusions have been drawn from recent reviews and experimental studies in wheat and barley, emphasizing that de-acclimation is an actively regulated process rather than a reversal of acclimation [1, 23].

The results further extend earlier hypotheses that de-acclimation may represent a recovery phase after stress [6]. In contrast to passive de-acclimation, which is associated with developmental transitions, our data indicate that active de-acclimation triggers a stress-like response in cold-acclimated wheat plants.

### Physiological basis of de-acclimation tolerance

**Fig. 16:**
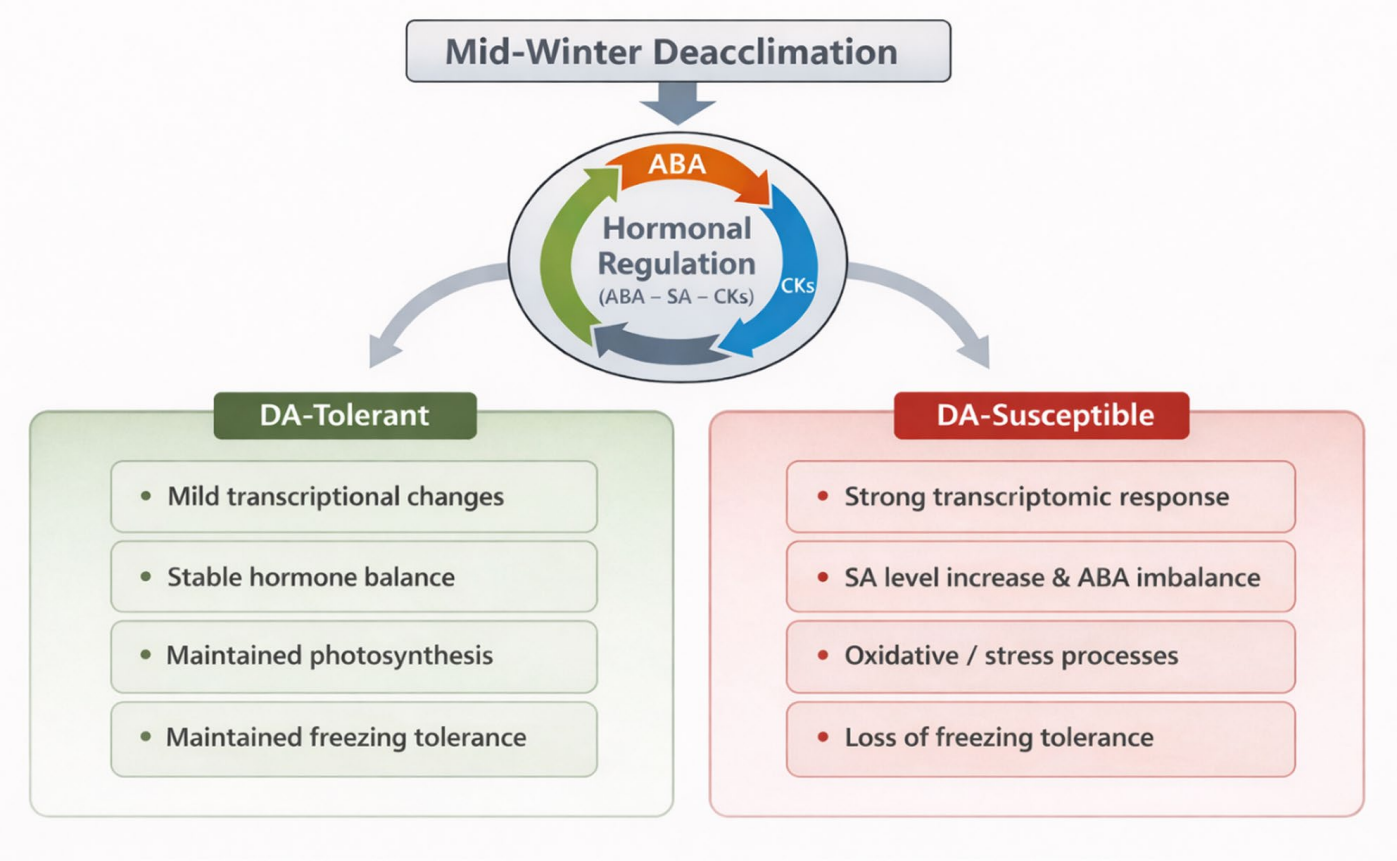
A graphical abstract summerising the obtained results regarding phenotypic, physiological, transcriptomic, and phytohormone-related responses to deacclimation in DA-tolerant and DA-susceptible wheat accessions

The phenotypic analyses revealed a clear distinction between tolerant and susceptible accessions in their ability to survive freezing after de-acclimation. While tolerant genotypes maintained high survival rates, susceptible lines showed complete loss of freezing tolerance (Fig. 16).

This supports the concept that freezing tolerance after de-acclimation is an independent trait, as previously suggested for cereals [5, 13].

Chlorophyll fluorescence measurements and principal component analysis further indicated that the physiological basis of freezing tolerance differs between cold-acclimated and de-acclimated states. Similar observations have been reported in wheat and other species, where de-acclimation is associated with changes in photosynthetic efficiency and energy flux parameters [13].

Recent physiological studies in wheat have also shown that de-acclimation leads to rapid metabolic adjustments accompanied by a decline in freezing tolerance, supporting the interpretation of our results [23].

### Transcriptomic response: stability versus extensive reprogramming

A key finding of this study is the contrasting scale of transcriptomic responses between tolerant and susceptible accessions. Susceptible genotypes exhibited a broader and more pronounced transcriptional reprogramming during de-acclimation, whereas tolerant accessions showed a more moderate response (Fig. 16).

This pattern suggests that tolerance to de-acclimation may be associated with the ability to maintain molecular stability during transient warming. In contrast, extensive transcriptional changes observed in susceptible lines may reflect a loss of regulatory control and activation of stress-related pathways.

The significant predominance of common molecular processes during cold acclimation in tolerant accessions further indicates the importance of cold acclimation mechanisms for subsequent de-acclimation tolerance.

This is in agreement with previous findings suggesting that the efficiency of cold acclimation determines plant performance during de-acclimation [13, 14].

### Hormonal regulation of de-acclimation response

The transcriptomic and hormonal analyses indicate that phytohormone signalling is a central component of the de-acclimation response. In particular, ABA, ethylene, and selected cytokinins were associated with differences between tolerant and susceptible accessions.

#### ABA-related regulation

The expression pattern of ABA hydroxylase and the observed changes in ABA levels suggest that ABA metabolism plays a key role in regulating the response to warming. Tolerant accessions maintained a more stable ABA-related response, whereas susceptible lines showed stronger fluctuations.

These observations are consistent with previous studies demonstrating that disturbances in hormone balance affect freezing tolerance after de-acclimation [8, 9].

Recent work has further highlighted the importance of ABA metabolism during de-acclimation, indicating that dynamic regulation of ABA levels is critical under fluctuating temperatures [24].

#### Ethylene signalling

Genes related to ethylene perception were strongly enriched among differentially expressed transcripts during de-acclimation. This suggests that ethylene signalling plays a central role in coordinating plant responses to warming.

Ethylene-related pathways have previously been linked to stress responses and developmental regulation, and their involvement in de-acclimation indicates integration of these processes.

#### Cytokinins and salicylic acid

Changes in cytokinin profiles and salicylic acid levels further indicate that hormonal balance plays an important role in differentiating tolerant and susceptible accessions.

Previous studies have shown that hormonal interactions influence plant responses to de-acclimation in different species [10].

Together, these findings support the concept that coordinated regulation of multiple hormone pathways, rather than changes in individual hormones, determines tolerance to de-acclimation.

### Wheat-specific insights in comparison with other species

Most previous studies on de-acclimation have focused on model species or barley [1, 4]. While some general mechanisms appear to be conserved, our results highlight several aspects that may be specific to wheat.

In particular, the strong association between ABA hydroxylase expression and tolerance, as well as the contrasting scale of transcriptomic responses between tolerant and susceptible accessions, point to wheat-specific regulatory mechanisms.

These findings suggest that extrapolation from other species may not fully capture the complexity of de-acclimation in wheat.

### Implications for breeding and climate resilience

The results of this study have direct implications for breeding strategies aimed at improving winter survival under climate change conditions. De-acclimation has already been identified as a key factor determining winter survival in cereals [3, 13].

The independence of freezing tolerance in cold-acclimated and de-acclimated states indicates that both traits should be considered simultaneously in selection programmes. The identification of hormone-related pathways, particularly those involving ABA and ethylene, provides potential molecular targets for improving tolerance.

Given the increasing frequency of mid-winter warm spells, improving tolerance to active de-acclimation will be essential for maintaining yield stability in winter wheat.

## Conclusions

In summary, this study confirms that, as previously shown in barley, active de-acclimation in winter wheat is not simply the inverse of cold acclimation, but represents a distinct and actively regulated process. Susceptible accessions exhibited substantially broader transcriptomic changes in response to de-acclimation than tolerant ones. This supports the hypothesis that tolerance to de-acclimation is associated with a limited or more controlled response to temperature increase, rather than with the activation of additional stress-related pathways.

A considerable proportion of genes differentially expressed during de-acclimation were related to stress and defence responses, which is consistent with observations in barley and other herbaceous species. In wheat, however, particular importance appears to be associated with hormone-related pathways. Both gene expression patterns and hormone profiling indicate that plant hormones, especially abscisic acid (ABA) and salicylic acid (SA), play a central role in shaping the response to active de-acclimation.

The results further suggest that the efficiency of cold acclimation may determine the ability of plants to maintain tolerance during subsequent warming periods. This highlights the importance of considering cold acclimation and de-acclimation responses jointly.

Taken together, these findings provide new insight into the molecular and physiological basis of de-acclimation tolerance in wheat and indicate that this trait should be considered an important target in breeding programmes aimed at improving resilience to increasingly variable winter conditions.

## Materials and methods

### Plant materials

Four accessions tolerant to active de-acclimation (STH 97, STH 14222, Plejada, and Wilejka) and four susceptible to it (DC 17 1216, POB 47817, PHR 2022/6, and SMH 334) were used in this study. Seeds were provided by Polish plant breeding companies: Hodowla Roślin Strzelce, Grupa IHAR, sp. z o.o. (both STH breeding lines and both commercial cultivars), Danko Hodowla Roślin sp. z o.o. (DC 17 1216), Małopolska Hodowla Roślin sp. z o.o. (POB 47817), Poznańska Hodowla Roślin sp. z o.o. (PHR 2022/6) and Hodowla Roślin Smolice, Grupa IHAR, sp. z o.o. (SMH 334), and represented both advanced breeding lines, and registered cultivars. The eight wheat accessions used in this study (four tolerant and four sensitive to active de-acclimation) were selected based on a three-year phenotypic evaluation of nearly 300 genotypes, including commercial cultivars used as reference controls, conducted within a broader project on frost tolerance under cold acclimation and de-acclimation conditions.

### Phenotyping of de-acclimation tolerance

The seeds of analysed accessions were sown in plastic boxes (30 × 39 cm) filled with a mixture of sand and garden soil 1:1 in 13 rows per box, each row with ten seeds corresponding to one accession. There were three boxes with the same accessions sown in different order to ensure the lowest environmental bias possible – making it 30 seeds sown per each accession, per treatment (freezing tolerance assessment of cold-acclimated plants, and freezing tolerance assessment of de-acclimated plants). The boxes were transferred to a growth chamber after sowing and kept in darkness at 25/17°C). Light was turned on after the plantlets started to emerge (irradiance of 400 µmol m-2s-1 (HPS lamps, SON-T+ AGRO, Philips, Brussels, Belgium), photoperiod of 10/14 h, day/night)). The temperature was lowered to 12 °C (day and night) and the irradiance was lowered to 250 µmol m-2s-1 eight days after sowing. The photoperiod of 10/14 h remained unchanged. 21 days after sowing, the plants were cold acclimated (3 weeks, 4/2°C, day/night temperature; photoperiod of 10/14 h and irradiance of 250 µmol m-2s-1). Cold acclimation was followed by 7 days of de-acclimating conditions, mimicking a mid-winter warm spell: 12/5°C, day/night temperature; photoperiod of 10/14 h and irradiance of 250 µmol m-2s-1).

After de-acclimation, the plants were sampled for freezing tolerance assessment. Survival of the plants after freezing was assessed using two methods: regrowth after freezing (FT-R, similar to [25], and measurements of chlorophyll fluorescence transient parameters (Table S1) after freezing [26]. For chlorophyll fluorescence measurements, leaves were excised about 1 cm above the soil immediately before freezing. The excised leaves were frozen under the same controlled conditions as the intact plants used for regrowth assessment, allowing direct comparison of freezing-induced damage among genotypes. After measurements, the analysis of chlorophyll fluorescence induction curve (OJIP) were performed and parameters describing energy flows and efficiencies for different steps of photosynthetic energy conversion were calculated [27, 28]. Two chlorophyll fluorescence parameters were selected as the best in terms of assessing de-acclimation tolerance (based on the results from [5] and ongoing analyses on barley and wheat): RC/CS_m_ (density of reaction centres capable of reducing plastoquinone (QA) at time t_max_) and PI_CSm_ (overall performance index in excited PSII in relation to the photosynthesising surface area of the sample (CS)). We acknowledge that leaf excision may itself trigger stress responses; therefore, the fluorescence measurements were interpreted here as a standardized comparative indicator of post-freezing injury, rather than as a direct measure of intact-plant photosynthetic performance.

Plastic boxes (containing the rest of the plants after cutting) were put in a programmed freezer. The temperature decreased from 0 °C to -12 °C at the rate of 2° C/h and after 12 h increased at the same rate. After freezing, the plants were transferred to a vegetation chamber at + 8 °C for 24 h (photoperiod: 10/14 h (day/night), irradiance: 250 µmol-m-2-s-1) and then to an unheated greenhouse (temperature around 15 °C). Plant survival rate was assessed by counting regrowth after freezing of de-acclimated plants. Re-growing plants were counted after 10 days and live plants after three weeks. Freezing tolerance was expressed as a percentage of regrowth (percentage of plants that resumed growth after freezing) verified by their survival rate (percentage of initial number of seedlings). Studies of freezing tolerance by means of chlorophyll fluorescence measurements were performed with the method described before, using Handy PEA fluorimeter (Hansa tech, Kings Lynn, UK) [27, 28].

### Cell membrane integrity measurements

Cell membrane integrity was assessed by conductometric measurement of electrolyte leakage using a CC-317 conductometer (Elmetron). At each sampling point (CA-0, CA-21, and DA-28), leaf fragments were excised from plants and immediately placed in scintillation vials containing deionized water (two fragments per vial; 10 vials representing 10 biological replicates for each of the eight wheat accessions).

The vials containing leaf tissues were then subjected to controlled freezing treatments. The temperature was decreased from 0 °C to the target temperature at a rate of 3 °C h⁻¹ to ensure ice nucleation. Depending on the sampling point, different sets of sub-zero temperatures were applied: −3, − 6, and − 9 °C for CA-0; −12, − 15, and − 18 °C for CA-21; and − 6, −9, and − 12 °C for DA-28. Leaf samples were maintained at each target temperature for 2 h.

Following freezing treatment, an additional volume of redistilled water was added to each vial, and samples were incubated on a shaker at room temperature for 24 h. Subsequently, the initial electrolyte leakage (EL1) was measured. To determine total electrolyte content, the samples were then immersed in liquid nitrogen to completely disrupt cell membranes, returned to the same vials, and shaken for a further 24 h, after which the final conductivity (EL2) was measured.

All measurements were corrected for the conductivity of deionized water (W), and membrane injury was expressed as electrolyte leakage (EL, %) = (EL1 − W) / (EL2 − W) × 100. Based on the results obtained at three freezing temperatures, the tEL50 parameter was calculated for each accession at each sampling point.

### Statistical treatment

Statistical analyses of the phenotyping data were performed using Statistica 13 PL software (StatSoft, Tulsa OK). The TEL50 for each accession and time point tEL50 values were estimated using the regression module, based on a linear regression fitted to the three electrolyte leakage points measured at three freezing temperatures.

Principal component analysis (PCA) was performed in Statistica software using the Principal Components & Classification Analysis (PCCA) procedure from the Multivariate Exploratory Techniques module. The analysis was based on the covariance matrix and was conducted on a set of mean-centered chlorophyll fluorescence (OJIP) parameters measured after freezing in cold-acclimated and de-acclimated samples in order to visualize multivariate relationships among accessions and treatments.

### RNA isolation

The seeds of plants for RNA sequencing were sown in plastic rectangular pots filled with a mixture of sand and garden soil 1:1. Each pot contained 10–12 plants. The conditions for plant growth, cold-acclimation and de-acclimation were the same as described for the phenotyping experiment. Wheat leaves weighing 0.05–0.09 g were sampled at three timepoints: just before cold-acclimation (CA-0), after cold-acclimation (CA-21), and after de-acclimation (DA-28), with three biological replicates per timepoint, and immediately frozen in liquid nitrogen. A total of 72 frozen leaf samples were collected from 8 accessions, across 3 biological replicates and 3 time-points. These samples were subjected to RNA isolation using the combination of Pure Link RNA Mini Kit (Invitrogen, Thermofisher Scientific, Waltham, MA, USA) and spin columns from RNeasy Plant Mini Kit (Qiagen, Hilden, Germany). The quantity and purity of RNA was checked using an UV-Vis Q5009 spectrophotometer (Quawell, San Jose, CA, USA). RNA integrity was tested using a 2100 Bioanalyzer (Agilent Technologies, Santa Clara, CA, USA).

### RNA sequencing and differential expression analysis

Total RNA samples were sent to weSEQ.IT company [29] for sequencing on Illumina NovaSeq 6000 platform in PE150 bp mode (Illumina, San Diego, CA, USA).

Sequencing data were checked for read quality using the FastQC tool [30] and stripped of adapters using the TrimGalore program [31]. The sequences were mapped to the wheat genome iwgsc_refseqv1.0 [32] using the Hisat2 v.2.1.0 program [33]. The next step was to analyze differentially expressed genes (DEGs) with DESeq2 v.1.24.0 [34] using R v.4.4.0 software [35]. A gene was considered to be significantly differentially expressed when the Benjamini-Hochberg false-discovery rate (FDR)-corrected P-value [36] was ≤ 0.01. Only two-fold or greater differences in expression of a given gene (1 ≤ log_2_FC ≤ -1) were considered.

Differences in expression for a given accession were tested between all time-points, averaging the results for three biological replicates. This yielded 24 DEG lists: for eight accessions, genes expressed before cold-acclimation (CA-0) were compared with genes expressed after cold-acclimation (CA-21) and after de-acclimation (DA-28). Genes from the DA-28 time-point were also compared with transcripts from the CA-21 time point. In addition, common genes undergoing expression in all tolerant accessions were compared with genes undergoing expression in each sensitive accession - at each of the three time-points - thus yielding an additional 3 DEG lists describing expression that depends on the level of tolerance of active de-acclimation, rather than on a change in external conditions.

We performed a series of comparisons of the resulting sets of DEGs, visualised as Venn diagrams using a web application [37].

To obtain information on which DEGs are specifically associated only with de-acclimation and are not the same as the reverse of the cold acclimation process, the common DEGs between DA-28 and CA-21 were compared (at *p* ≤ 0.01) with the common DEGs between CA-0 and CA-21. The resultant de-acclimation-specific DEGs were then contrasted in tolerant and sensitive subjects.

In order to further investigate the mechanism of tolerance of active de-acclimation in winter wheat, the DEGs specific only to the de-acclimation process present in tolerant accessions (i.e. also those common to tolerant and sensitive accessions) obtained from previous comparisons were compared with the DEGs for tolerant versus sensitive accessions present only at the DA-28 time point. The comparison was performed at *p* ≤ 0.01.

### Gene ontology analysis and annotation

For the obtained DEGs associated with active de-acclimation in winter wheat at *p* ≤ 0.01, a ‘GO enrichment’ gene ontology analysis was performed using the GOSt tool in the Gprofiler application [38]. The analysis was performed for common wheat, for all known genes using a dedicated ‘g: SCS threshold’ false-positive correction method, similar to the Bonferroni correction [39] and FDR < 0.05.

Gene annotation was conducted using the annotations in the wheat genome [32] and by performing BLAST searches against similar sequences in other organisms. Sequences of interest were analysed using the Omics Box 3.2.6 application [40], in the ‘functional analysis’ module, with Blastp [20] and/or Blastp-fast [41] under default settings. An ‘InterProScan’ analysis [42] was also performed and the ‘gene ontology’ analysis (GO annotation) was repeated and combined (GO mapping).

Annotated genes were analysed in terms of functions represented and those for which functions and processes with a definable role were significantly overrepresented in the GO analysis, as well as additional genes related to the stress response, were selected. Heatmap plots were produced for these selected groups using the ‘heatmaply’ function in the R package [35]. For the genes associated with the de-acclimation process (i.e. the change between the CA-21 and DA-28 time points), the log_2_FC expression change values for DA-28/CA-21 were used to produce heatmap plots, while for the other genes expressed at DA-28, the TPM (Transcripts per Million [43]) values were used to compare the expression of a given gene in each accession. The genes with most distinct changes between tolerant and susceptible accessions were selected for the verification via RT-qPCR.

### Analysis of the expression of selected genes

The sequences of seven genes selected for the verification of the RNAseq results were downloaded from the EnsemblPlants.org database [21, 44], and an additional gene *TraesCS4A02G111500* [20]. The primer 3 plus website was used to design the primers and probe sequences for the selected genes [45, 46] and a list of the primers designed is attached in Table S2.

Plants were grown and exposed to cold acclimation and subsequent de-acclimation under identical conditions as detailed earlier. Leaf samples were collected at three distinct time points—CA-0 (prior to cold acclimation), CA-21 (after 21 days of cold acclimation), and DA-28 (following 28 days of de-acclimation). Three biological replicates per time point were used, each comprising leaves from a separate plant. Total RNA for gene expression profiling was extracted from leaf tissues following the method described above.

Complementary DNA (cDNA) was synthesized from the isolated RNA using the QuantiTect Reverse Transcription Kit (Qiagen). Quantitative real-time PCR (RT-qPCR) was employed to assess expression changes in selected target genes. The reactions were carried out using a QuantStudio 3 Real-Time PCR System (Thermo Fisher Scientific, Waltham, MA, USA). Gene amplification was monitored via fluorescence signals from SYBRGreen [47]. Two reference genes— *ACTIN2* [48] and *26 S RNA* [49] - were used for normalization. Each RT-qPCR assay was performed with three independent biological replicates per genotype, and each sample was analyzed in triplicate technical replicates. Reaction mixtures included 900 nM of each primer, approximately 35 ng of cDNA, and TaqMan™ Gene Expression Master Mix (Thermo Fisher Scientific). Relative gene expression levels were determined using a modified standard curve approach [50]. The expression level at the CA-0 time point—normalized against the geometric mean of the two reference genes—served as the baseline (expression value = 1) for comparative analysis across all time points. Geometric means were calculated from three technical replicates × three biological replicates × two reference genes, and standard errors were estimated from log-transformed replicate data.

### Phytohormone extraction and quantification

The entire aboveground part of the plant (leaves and stems)was sampled in three timepoints – CA-0, CA-21 and DA-28 and rapidly cryopreserved in liquid nitrogen immediately following sampling. One plant constituted one biological replicate, each accession in each timepoint consisted of 3–5 replicates. The samples were subsequently lyophilized and pulverized, then extracted in a chilled extraction solvent composed of methanol, water, and formic acid (15:4:1, v/v/v), following the protocol of Dobrev and Kamínek [51]. To ensure accurate quantification, a mixture of internal standards—including deuterium-labeled indole-3-acetic acid (D_5_-IAA), salicylic acid (D_4_-SA) and abscisic acid (D_6_-ABA), as well as ¹⁵N-labeled trans-zeatin was added during extraction.

Extracts were subjected to solid-phase extraction (SPE) using Oasis MCX columns (Waters, USA), resulting in two distinct fractions: an “acidic” fraction for IAA, SA and ABA, eluted with pure methanol [52]; a “basic” fraction containing cytokinin, eluted with a methanolic ammonia solution [53].

The acidic fraction, containing IAA, SA and ABA, was dried under vacuum and reconstituted in 100 µL of methanol for analysis. Hormonal quantification was performed on a Supelco Ascentis RP-Amide column (7.5 cm × 2.1 mm, 2.7 μm) using a binary gradient system: solvent A consisted of 0.1% formic acid in water, while solvent B was a 1:1 mixture of acetonitrile and methanol. The gradient elution profile started at 20% B, increased to 80% at 8 min, and returned to 20% by 3.5 min, with a total run time of 11.5 min. Chromatographic separation and detection were conducted using an Agilent Technologies 1260 Infinity HPLC coupled to an Agilent 6410 Triple Quadrupole LC/MS system equipped with ESI.

For each analyte, two mass transitions were monitored in Multiple Reaction Monitoring (MRM) mode: the most abundant ion was used for quantification, and the second-most one served as a qualifier ion. Data acquisition and instrument control were carried out using Agilent Mass Hunter Workstation software (version 5).

Cytokinin species—including trans-zeatin (t-Z), cis-zeatin (c-Z) [54], dihydro-zeatin (dh-Z), their riboside forms (t-ZR, c-ZR), kinetin riboside (KinR), meta-topolin, orto-topolin and isopentenyladenine (iPA) -were isolated from the basic SPE fraction. These were grouped into total measured cytokinin content (CKs), with Z-type cytokinin comprising t-Z, c-Z, t-ZR, and c-ZR.

After removal of acidic analytes, the SPE columns were washed with 0.35 M aqueous ammonia to remove contaminants, followed by elution of cytokinin compounds using 0.35 M ammonia in 60% methanol [53]. The eluate was dried, reconstituted in 100 µL methanol, and subjected to chromatographic analysis using the same HPLC setup as described for IAA, SA and ABA.

Cytokinin analysis employed a solvent system of water containing 0.001% acetic acid (solvent A) and acetonitrile with 0.001% acetic acid (solvent B), operated at a constant flow rate of 1.5 mL min⁻¹. The gradient profile was as follows: 2.5% B at 0 min, 10% at 3 min, 25% at 9 min, 75% at 16 min, returning to 2.5% at 24.5 min. MRM transitions for the most prominent secondary ions were monitored.

## Supplementary Information

Supplementary Material 1.

Supplementary Material 2.

Supplementary Material 3.

Supplementary Material 4.

Supplementary Material 5.

## Data Availability

The datasets generated and/or analysed during the current study are available from public databases. Raw RNAseq data and TPM processed file are available from ArrayExpress (E-MTAB-16628), while the trimmed RNAseq data (without adapters) and chlorophyll fluorescence data are available from University of Agriculture in Krakow repository (DOI: 10.15576/REPOURK/2026.1.2); link to download the research data: https://repo.ur.krakow.pl/info/researchdata/URK24b5d4af4d5f498db36cc9982d5515fb/.

## References

[1] Kosová K, Nešporová T, Vítámvás P, Vítámvás J, Klíma M, Ovesná J, et al. How to survive mild winters: Cold acclimation, deacclimation, and reacclimation in winter wheat and barley. Plant Physiol Biochem. 2025;220:109541. 10.1016/J.PLAPHY.2025.109541. 39862458 10.1016/j.plaphy.2025.109541

[2] Rapacz M, Ergon Å, Höglind M, Jørgensen M, Jurczyk B, Østrem L, et al. Overwintering of herbaceous plants in a changing climate. Still more questions than answers. Plant Sci. 2014;225:34–44. 10.1016/J.PLANTSCI.2014.05.009. 25017157 10.1016/j.plantsci.2014.05.009

[3] Rapacz M, Macko-Podgórni A, Jurczyk B, Kuchar L. Modeling wheat and triticale winter hardiness under current and predicted winter scenarios for Central Europe: A focus on deacclimation. Agric For Meteorol. 2022;313:108739. 10.1016/J.AGRFORMET.2021.108739.

[4] Stachurska J, Sadura-Berg I, Rys M. When Warm Breaks Cold: Understanding Deacclimations and Reacclimations Cycles as a Key to Winter Crop Resilience. Int J Mol Sci 2025. 2025;26:26. 10.3390/IJMS262211080. 10.3390/ijms262211080 PMC12652565 41303561

[5] Wójcik-Jagła M, Rapacz M. Freezing tolerance and tolerance to de-acclimation of European accessions of winter and facultative barley. Sci Rep. 2023;13:1–11. 10.1038/S41598-023-47318-Y;SUBJMETA. 37968280 10.1038/s41598-023-47318-y PMC10651919

[6] Wójcik-Jagła M, Daszkowska-Golec A, Fiust A, Kopeć P, Rapacz M. Identification of the genetic basis of response to de-acclimation in winter barley. Int J Mol Sci. 2021;22:1–33. 10.3390/IJMS22031057. 10.3390/ijms22031057 PMC7865787 33494371

[7] Kalberer SR, Wisniewski M, Arora R. Deacclimation and reacclimation of cold-hardy plants: Current understanding and emerging concepts. Plant Sci. 2006;171:3–16. 10.1016/J.PLANTSCI.2006.02.013.

[8] Stachurska J, Rys M, Pociecha E, Kalaji HM, Dąbrowski P, Oklestkova J, et al. Deacclimation-Induced Changes of Photosynthetic Efficiency, Brassinosteroid Homeostasis and BRI1 Expression in Winter Oilseed Rape (Brassica napus L.)—Relation to Frost Tolerance. Int J Mol Sci. 2022;23. 10.3390/IJMS23095224/S1. 10.3390/ijms23095224 PMC9102500 35563614

[9] Stachurska J, Janeczko A. Physiological and Biochemical Background of Deacclimation in Plants, with Special Attention Being Paid to Crops: A Minireview. Agronomy. 2024;14. 10.3390/AGRONOMY14030419.

[10] Pociecha E, Janeczko A, Dziurka M, Gruszka D. Disturbances in the Biosynthesis or Signalling of Brassinosteroids That Are Caused by Mutations in the HvDWARF, HvCPD and HvBRI1 Genes Increase the Tolerance of Barley to the Deacclimation Process. J Plant Growth Regul. 2020;39:1625–37. 10.1007/S00344-020-10183-4.

[11] Chen F, He J, Jin G, Chen ZH, Dai F. Identification of novel microRNAs for cold deacclimation in barley. Plant Growth Regul. 2020;92:389–400. 10.1007/S10725-020-00646-9.

[12] Rys M, Pociecha E, Oliwa J, Ostrowska A, Jurczyk B, Saja D et al. Deacclimation of Winter Oilseed Rape—Insight into Physiological Changes. Agronomy 2020, Vol 10, Page 1565. 2020;10:1565. 10.3390/AGRONOMY10101565

[13] Juszczak I, Cvetkovic J, Zuther E, Hincha DK, Baier M. Natural variation of cold deacclimation correlates with variation of cold-acclimation of the plastid antioxidant system in Arabidopsis thaliana accessions. Front Plant Sci. 2016;7:180268. 10.3389/FPLS.2016.00305/BIBTEX. 10.3389/fpls.2016.00305 PMC4794505 27014325

[14] Zuther E, Juszczak I, Ping Lee Y, Baier M, Hincha DK. Time-dependent deacclimation after cold acclimation in Arabidopsis thaliana accessions. Sci Rep. 2015;5:1–10. 10.1038/SREP12199;SUBJMETA. 10.1038/srep12199 PMC4648415 26174584

[15] Vaitkevičiūtė G, Aleliūnas A, Brazauskas G, Armonienė R. Deacclimation and reacclimation processes in winter wheat: novel perspectives from time-series transcriptome analysis. Front Plant Sci. 2024;15. 10.3389/FPLS.2024.1395830. 10.3389/fpls.2024.1395830 PMC11130478 38807787

[16] Rapacz M, Jurczyk B, Sasal M. Deacclimation may be crucial for winter survival of cereals under warming climate. Plant Sci. 2017;256:5–15. 10.1016/J.PLANTSCI.2016.11.007. 28167038 10.1016/j.plantsci.2016.11.007

[17] Bartczak A, Kaczmarek H, Badocha M, Krzeminski M, Tyszkowski S. Measured and predicted freeze-thaw days frequencies in climate change conditions in central Poland. PeerJ. 2021;9:e12153. 10.7717/PEERJ.12153/SUPP-1. 34754616 10.7717/peerj.12153 PMC8555500

[18] IPCC. IPCC, 2023: Climate Change 2023: Synthesis Report. Contribution of Working Groups I, II and III to the Sixth Assessment Report of the Intergovernmental Panel on Climate Change [Core Writing Team, H. Lee and J. Romero, editors]. IPCC, Geneva, Switzerland. 2023. 10.59327/IPCC/AR6-9789291691647

[19] An C, Gao Y. Essential Roles of the Linker Sequence Between Tetratricopeptide Repeat Motifs of Ethylene Overproduction 1 in Ethylene Biosynthesis. Front Plant Sci. 2021;12:657300. 10.3389/FPLS.2021.657300/BIBTEX. 33936142 10.3389/fpls.2021.657300 PMC8081955

[20] BLAST. Basic Local Alignment Search Tool. https://blast.ncbi.nlm.nih.gov/Blast.cgi. Accessed 25 Oct 2025.

[21] Howe KL, Contreras-Moreira B, De Silva N, Maslen G, Akanni W, Allen J, et al. Ensembl Genomes 2020—enabling non-vertebrate genomic research. Nucleic Acids Res. 2020;48:D689–95. 10.1093/NAR/GKZ890. 31598706 10.1093/nar/gkz890 PMC6943047

[22] https://www.uniprot.org/. BTB domain-containing protein - Triticum aestivum (Wheat) | UniProtKB | UniProt. https://www.uniprot.org/uniprotkb/A0A9R1K630/entry. Accessed 22 Oct 2025.

[23] Vaitkevičiūtė G, Aleliūnas A, Gibon Y, Armonienė R. The effect of cold acclimation, deacclimation and reacclimation on metabolite profiles and freezing tolerance in winter wheat. Front Plant Sci. 2022;13:959118. 10.3389/fpls.2022.959118. 36046584 10.3389/fpls.2022.959118 PMC9421140

[24] Rys M, Bocianowski J, Dziurka M, Jurczyk B, Stachurska J, Waligórski P, et al. Hormonal Balance in Relation to Expression of Selected Genes Connected with Hormone Biosynthesis and Signalling—The Effect of Deacclimation Process in Oilseed Rape. Int J Mol Sci. 2025;26:7408. 10.3390/IJMS26157408/S1. 40806537 10.3390/ijms26157408 PMC12347709

[25] Larsen A. Larsen: Freezing tolerance in grasses. Methods for… Google Scholar. 1978.https://scholar.google.com/scholar_lookup?&title=Freezing+tolerance+in+grasses+-+methods+for+testing+in+controlled+environments%2E&journal=Meld%2E+Norg%2E+Landbruks%2E&author=Larsen+A.&publication_year=1978&volume=57&pages=2-56#d=gs_cit&t=1761301159722&u=%2Fscholar%3Fq%3Dinfo%3AYamI0DxaqhkJ%3Ascholar.google.com%2F%26output%3Dcite%26scirp%3D0%26hl%3Den.Accessed 24 Oct 2025.

[26] Tyrka M, Rapacz M, Fiust A, Wójcik-Jaglła M. Quantitative trait loci mapping of freezing tolerance and photosynthetic acclimation to cold in winter two- and six-rowed barley. Plant Breeding. 2015;134:271–82. 10.1111/PBR.12270.

[27] Østrem L, Rapacz M, Larsen A, Marum P, Rognli OA. Chlorophyll a Fluorescence and Freezing Tests as Selection Methods for Growth Cessation and Increased Winter Survival in ×Festulolium. Front Plant Sci. 2018;9:1200. 10.3389/FPLS.2018.01200. 30177939 10.3389/fpls.2018.01200 PMC6109792

[28] Rapacz M, Sasal M, Kalaji HM, Kos̈cielniak J. Is the OJIP test a reliable indicator of winter hardiness and freezing tolerance of common wheat and triticale under variable winter environments? PLoS ONE. 2015;10. 10.1371/JOURNAL.PONE.0134820. 10.1371/journal.pone.0134820 PMC4521754 26230839

[29] Rybnik P. https://weseq.it/- Google Search. https://www.google.com/search?q=(Rybnik%2C+Poland%2C+https%3A%2F%2Fweseq.it%2F)&oq=(Rybnik%2C+Poland%2C+https%3A%2F%2Fweseq.it%2F)&gs_lcrp=EgZjaHJvbWUyBggAEEUYOTIHCAEQABjvBTIHCAIQABjvBTIHCAMQABjvBTIHCAQQABjvBdIBCDEyMDFqMGo3qAIIsAIB8QV3z_0fI42uZPEFd8_9HyONrmQ&sourceid=chrome&ie=UTF-8. Accessed 31 Oct 2025.

[30] Babraham Bioinformatics - FastQC. Babraham Bioinformatics - FastQC A Quality Control tool for High Throughput Sequence Data. 2018. https://www.bioinformatics.babraham.ac.uk/projects/fastqc/. Accessed 31 Oct 2025.

[31] Martin M. Cutadapt removes adapter sequences from high-throughput sequencing reads. EMBnet J. 2011;17:10–2. 10.14806/EJ.17.1.200.

[32] (IWGSC) TIWGSC et al, et al. Shifting the limits in wheat research and breeding using a fully annotated reference genome. New York, NY: Science; 2018. p. 361. 10.1126/SCIENCE.AAR7191. 10.1126/science.aar7191 30115783

[33] Kim D, Paggi JM, Park C, Bennett C, Salzberg SL. Graph-based genome alignment and genotyping with HISAT2 and HISAT-genotype. Nat Biotechnol. 2019;37:907–15. 10.1038/S41587-019-0201-4;SUBJMETA. 31375807 10.1038/s41587-019-0201-4 PMC7605509

[34] Love MI, Huber W, Anders S. Moderated estimation of fold change and dispersion for RNA-seq data with DESeq2. Genome Biol. 2014;15. 10.1186/S13059-014-0550-8. 10.1186/s13059-014-0550-8 PMC4302049 25516281

[35] R Core Team, Core Team R. R (2024) A Language and Environment for Statistical Computing. R Foundation for Statistical Computing. - References - Scientific Research Publishing. 2024. https://www.scirp.org/reference/referencespapers?referenceid=3864327. Accessed 24 Oct 2025.

[36] Benjamini Y, Hochberg Y. Controlling the False Discovery Rate: A Practical and Powerful Approach to Multiple Testing. J Roy Stat Soc: Ser B (Methodol). 1995;57:289–300. 10.1111/J.2517-6161.1995.TB02031.X.

[37] Oliveros JC, Venny. An interactive tool for comparing lists with Venn’s diagrams. 2007. https://bioinfogp.cnb.csic.es/tools/venny/. Accessed 31 Oct 2025.

[38] g:Profiler. – a web server for functional enrichment analysis and conversions of gene lists. https://biit.cs.ut.ee/gprofiler/gost. Accessed 25 Oct 2025. 10.1093/nar/gkz369 PMC6602461 31066453

[39] Weisstein EW. Bonferroni Correction -- from Wolfram MathWorld. https://mathworld.wolfram.com/BonferroniCorrection.html. Accessed 25 Oct 2025.

[40] OmicsBox. 3.2.6 0. Bioinformatics Software OmicsBox | Biobam. https://www.biobam.com/omicsbox/. Accessed 25 Oct 2025.

[41] Shiryev SA, Papadopoulos JS, Schäffer AA, Agarwala R. Improved BLAST searches using longer words for protein seeding. Bioinf (Oxford England). 2007;23:2949–51. 10.1093/BIOINFORMATICS/BTM479. 10.1093/bioinformatics/btm479 17921491

[42] InterProScan. InterProScan - InterPro. https://www.ebi.ac.uk/interpro/search/sequence/. Accessed 25 Oct 2025.

[43] Zhao Y, Li MC, Konaté MM, Chen L, Das B, Karlovich C, et al. TPM, FPKM, or Normalized Counts? A Comparative Study of Quantification Measures for the Analysis of RNA-seq Data from the NCI Patient-Derived Models Repository. J Translational Med. 2021;19. 10.1186/S12967-021-02936-W. 10.1186/s12967-021-02936-w PMC8220791 34158060

[44] Ensembl Plants. Ensembl Plants. https://plants.ensembl.org/index.html. Accessed 24 Oct 2025.

[45] Primer3Plus - Pick Primers. https://www.primer3plus.com/index.html. Accessed 18 Oct 2025.

[46] Untergasser A, Cutcutache I, Koressaar T, Ye J, Faircloth BC, Remm M, et al. Primer3—new capabilities and interfaces. Nucleic Acids Res. 2012;40:e115–115. 10.1093/NAR/GKS596. 22730293 10.1093/nar/gks596 PMC3424584

[47] Bustin SA. Absolute quantification of mRNA using real-time reverse transcription polymerase chain reaction assays. J Mol Endocrinol. 2000;25:169–93. 10.1677/JME.0.0250169. 11013345 10.1677/jme.0.0250169

[48] Wang SB, Feng JY, Ren WL, Huang B, Zhou L, Wen YJ, et al. Improving power and accuracy of genome-wide association studies via a multi-locus mixed linear model methodology. Sci Rep. 2016;6. 10.1038/srep19444. 10.1038/srep19444 PMC4726296 26787347

[49] Ali-Benali MA, Alary R, Joudrier P, Gautier MF. Comparative expression of five Lea Genes during wheat seed development and in response to abiotic stresses by real-time quantitative RT-PCR. Biochimica et Biophysica Acta - Gene Structure and Expression. 2005;1730:56–65. 10.1016/J.BBAEXP.2005.05.011 10.1016/j.bbaexp.2005.05.011 16023228

[50] Pfaffl MW. A new mathematical model for relative quantification in real-time RT-PCR. Nucleic Acids Res. 2001;29:E45. 10.1093/NAR/29.9.E45. 11328886 10.1093/nar/29.9.e45 PMC55695

[51] Ivanov Dobrev P, Kamínek M. Fast and efficient separation of cytokinins from auxin and abscisic acid and their purification using mixed-mode solid-phase extraction. J Chromatogr A. 2002;950:21–9. 10.1016/S0021-9673(02)00024-9. 11990994 10.1016/s0021-9673(02)00024-9

[52] Štefančič M, Štampar F, Veberič R, Osterc G. The levels of IAA, IAAsp and some phenolics in cherry rootstock ‘GiSelA 5’ leafy cuttings pretreated with IAA and IBA. Sci Hort. 2007;112:399–405. 10.1016/J.SCIENTA.2007.01.004.

[53] Żur I, Dubas E, Krzewska M, Waligórski P, Dziurka M, Janowiak F. Hormonal requirements for effective induction of microspore embryogenesis in triticale (× Triticosecale Wittm.) anther cultures. Plant Cell Rep. 2015;34:47–62. 10.1007/s00299-014-1686-4. 25261160 10.1007/s00299-014-1686-4 PMC4282712

[54] Gajdošová S, Spíchal L, Kamínek M, Hoyerová K, Novák O, Dobrev PI, et al. Distribution, biological activities, metabolism, and the conceivable function of cis-zeatin-type cytokinins in plants. J Exp Bot. 2011;62:2827–40. 10.1093/JXB/ERQ457. 21282330 10.1093/jxb/erq457

