# Supplementary material for: Mid-winter de-acclimation triggers a stress-like response in leaves of cold-acclimated wheat (Triticum aestivum L.) at the tillering stage
Source: BMC Plant Biol. 2026 Jun 2;26:1318. doi: 10.1186/s12870-026-09102-8 (PMC13450400; doi:10.1186/s12870-026-09102-8)
Supplement: Supplementary file 1 [file 12870_2026_9102_MOESM1_ESM.docx]

**Table S1.** Formulae and glossary of terms used by the OJIP-test in the present study (modified after Strasser et al. 2004).

| *Data extracted from the recorded fluorescence transient OJIP* | |
| --- | --- |
| F_t_ | fluorescence at time t after onset of actinic illumination |
| F_50µs_ or F_20µs_ | minimal reliable recorded fluorescence, at 50 µs with the PEA-  or 20 µs with the Handy-PEA-fluorimeter |
| F_300µs_ | fluorescence intensity at 300µs |
| F_J_ ≡ F_2ms_ | fluorescence intensity at the J-step (2 ms) of OJIP |
| F_I_ ≡ F_30ms_ | fluorescence intensity at the I-step (30 ms) of OJIP |
| F_P_ | maximal recorded fluorescence intensity, at the peak P of OJIP |
| Fluorescence parameters derived from the extracted data | |
| F_0_ ≅ F_50µs_ or ≅ F_20µs_ | minimal fluorescence (all PSII RCs are assumed to be open) |
| F_M_ (= F_P_) | maximal fluorescence, when all PSII RCs are closed (equal to F_P_ when the actinic light intensity is above 500 μmol photons m^-2^ s^-1^  and provided that all RCs are active as Q_A_ reducing) |
| F_V_ ≡ F_M_ - F_0_ | maximal variable fluorescence |
| V_t_ ≡ F_υ_/F_V_ ≡ ( F_t_ - F_0_)/(F_M_ - F_0_) | relative variable fluorescence at time t |
| M_0_ ≡ [(ΔF/Δt)_0_]/(F_M_ - F_50µs_)  ≡ 4( F_300µs_-F_50µs_)/(F_M_- F_50µs_) | approximated initial slope (in ms^-1^) of the fluorescence transient normalised on the maximal variable fluorescence F_V_ |
| Specific energy fluxes (per Q_A_-reducing PSII reaction center - RC) | |
| ABS /RC = M_0_ (1/V_J_)(1/φ_Po_) | absorption flux (of antenna Chls) per RC |
| TR_0_/RC = M_0_ (1/V_J_) | trapped energy flux (leading to Q_A_ reduction) per RC |
| ET_0_/ RC = M_0_ (1/V_J_)ψ_Eo_ | electron transport flux (further than Q_A_^−^) per RC |
| DI_0_/RC = (ABS/RC) - (TR_0_/RC) | dissipated energy flux, per RC |
| Quantum yields and efficiencies | |
| φ_Po_ ≡ TR_0_/ABS = Fv/FM | maximum quantum yield for primary photochemistry |
| ψ_o_ ≡ ET_0_/TR_0_ = (1-V_J_) | efficiency/probability for electron transport (ET), i.e. efficiency/probability that an electron moves further than Q_A_^-^ |
| φ_Eo_ ≡ ET_0_/ABS = [1-(F_0_/F_M_)]ψ_Eo_ | quantum yield for electron transport (ET) |
| φ_Ro_ ≡ RE_0_/ABS = [1-(F_0_/F_M_)]ψ_Eo_ δ_Ro_ | quantum yield for reduction of end electron acceptors at the PSI acceptor side (RE) |
| *Phenomenological fluxes* | |
| ABS/CS = *F*o or ABS/CSM = *F*M  TR_0_/CS = ΦPo·(ABS/CS)  ET_0_/CS = ΦPo·Ψo·(ABS/CS)  DI_0_/CS = (ABS/CS) – (TRo/CS) | absorption per excited cross-section  trapping per excited cross-section  electron transport per excited cross-section  dissipated energy flux per excited cross-section |
| Performance indexes (products of terms expressing partial potentials at steps of energy bifurcations) | |
| PI_ABS_ ≡ [γ_RC_/(1-γ_RC_ )].[φ_Po_/(1- φ_Po_)].[ψ_o_/(1- ψ_o_] | performance index (potential) for energy conservation from  exciton to the reduction of intersystem electron acceptors |
| PI_total_ ≡ (PI_ABS_).(δ_Ro_/1- δ_Ro_) | performance index (potential) for energy conservation from  exciton to the reduction of PSI end acceptors |
| *Subscript “0” indicates that the parameter refers to the onset of illumination* | |
