# Supplementary material for: Mid-winter de-acclimation triggers a stress-like response in leaves of cold-acclimated wheat (Triticum aestivum L.) at the tillering stage
Source: BMC Plant Biol. 2026 Jun 2;26:1318. doi: 10.1186/s12870-026-09102-8 (PMC13450400; doi:10.1186/s12870-026-09102-8)
Supplement: Supplementary file 2 [file 12870_2026_9102_MOESM2_ESM.docx]

**Table S4.** Sequences of the primers used for the analysis of expression of the genes potentially involved in tolerance to de-acclimation selected on the basis of the RNAseq results.

| **Gene** | **Gene ID** | **Forward Primer 3′-5′** | **Reverse Primer 3′-5′** |
| --- | --- | --- | --- |
| *Licheninase* | *TraesCS1B02G249000* | GCCCGGATCTACAACCAGTA | GCTGCATGTTGGGGTAGAAG |
| *ABA hydroxylase* | *TraesCS6A02G271300* | TCCAAGAACCCCAACGTCTT | TTGAAGATGGGCCCGTACTT |
| *Gibberellin oxidase* | *TraesCS5D02G566200* | CTGTCGCTGGAGATCATGGA | TAGTTGAGGCGCATGATGGA |
| *phenylalanine ammonia-lyase (PAL)* | *TraesCS1D02G039400* | AAGTCAATCGAGCGGGAGAT | AAGTCGTTCACCAGCTCTGA |
| *PPR5* | *TraesCS6A02G303300* | TGGAGCAGGTGTTCAAGAGT | TGAGACACTCTTGCGTCACA |
| *Molybdate transporter* | *TraesCS2A02G496200* | CTGGGGCTGGTGAAGATAACA | TGCCAGAACCCGATCTTGAA |
| *WRKY* | *TraesCS6B02G175100* | TGCAAACCCGTCATCTCCAA | GTTGTCCTTGGTCACCTTCTG |
| *ETO1-like* | *TraesCS4A02G111500* | ACGCTGACTTACCCGTACAA | ATCCTGAAGCTGAGCACCTT |
