# Supplementary material for: Mid-winter de-acclimation triggers a stress-like response in leaves of cold-acclimated wheat (Triticum aestivum L.) at the tillering stage
Source: BMC Plant Biol. 2026 Jun 2;26:1318. doi: 10.1186/s12870-026-09102-8 (PMC13450400; doi:10.1186/s12870-026-09102-8)
Supplement: Supplementary file 3 [file 12870_2026_9102_MOESM4_ESM.docx]

**Fig.S1.**

Differentially expressed genes (DEGs) identified between non-acclimated (CA-0) and cold-acclimated (CA-21) wheat accessions. DEGs were defined as genes showing |log₂FC| ≥ 1 and P ≤ 0.01. The analysis was performed separately for accessions classified as tolerant or susceptible to active de-acclimation. Upregulated genes represent transcripts with higher expression in CA-21 compared to CA-0, whereas downregulated genes represent transcripts with lower expression in CA-21 relative to CA-0. Numbers indicate the total counts of DEGs in each category.

**Fig. S2.**

Differentially expressed genes (DEGs) identified between non-acclimated (CA-0) and de-acclimated (DA-28) wheat accessions. DEGs were defined as genes showing |log₂FC| ≥ 1 and P ≤ 0.01. The analysis was performed separately for accessions classified as tolerant or susceptible to active de-acclimation. Upregulated genes represent transcripts with higher expression in DA-28 compared to CA-0, whereas downregulated genes represent transcripts with lower expression in DA-28 relative to CA-0. Numbers indicate the total counts of DEGs in each category.

**Fig. S3.**

Comparison of differentially expressed genes (DEGs) distinguishing tolerant and susceptible wheat accessions at the de-acclimated stage (DA-28) and DEGs specifically associated with the de-acclimation process (DA-28 vs. CA-21). DEGs were defined as genes showing |log₂FC| ≥ 1 and P ≤ 0.01. The diagram illustrates the overlap between (i) genes differentially expressed between tolerant and susceptible accessions at DA-28 (reflecting genotype-dependent differences at the de-acclimated stage) and (ii) genes differentially expressed between DA-28 and CA-21 (reflecting changes induced by the de-acclimation process). Numbers indicate the size of each gene set and their intersection.

**Fig. S4**.

Heatmap of normalized gene expression levels (TPM) for selected genes at the de-acclimated stage (DA-28) in wheat accessions differing in tolerance to active de-acclimation. Rows represent individual genes, and columns represent individual accessions. The first four columns correspond to tolerant accessions, and the last four correspond to susceptible accessions. Color scale indicates relative expression levels (TPM), with higher and lower values represented by contrasting colors. Genes included in the analysis were selected based on functional relevance and/or differential expression patterns.
