# Supplementary material for: Mid-winter de-acclimation triggers a stress-like response in leaves of cold-acclimated wheat (Triticum aestivum L.) at the tillering stage
Source: BMC Plant Biol. 2026 Jun 2;26:1318. doi: 10.1186/s12870-026-09102-8 (PMC13450400; doi:10.1186/s12870-026-09102-8)
Supplement: Supplementary file 4 [file 12870_2026_9102_MOESM5_ESM.docx]

**Table S5.** Selected candidate genes for verification of expression levels. Blue - tolerant to active de-acclimation accessions, orange - sensitive accessions; relative expression level of log2FC DA-28/CA21. Expression level in RNAseq experiment: DA-28/CA-21 – upper value; expression level in qPCR verification experiment: DA-28/CA-21 – lower value.

| **Gene ID** | **Name** | **Expression level in DA-28/CA-21** | | | | | | | |
| --- | --- | --- | --- | --- | --- | --- | --- | --- | --- |
|  |  | **STH 97** | **STH 14222** | **Plejada** | **Wilejka** | **DC 17_1216** | **POB 47817** | **PHR 2022_6** | **SMH 334** |
| TraesCS6A 02G271300 | abscisic acid 8'-hydroxylase 1-like | 1.60  -0.17 | 1.50  0.06 | 1.70  -0.09 | 1.95  -0.22 | NA  -1.16 | -1.83  -1.07 | NA  -1.33 | NA  -1.09 |
| TRAESCS1B 02G249000 | lichenase-2 | 4.97  2.46 | 2.7  1.46 | 2.08  1.96 | 2.43  0.75 | 5.31  2.03 | 8.96  0.83 | 6.2  1.2 | 5.35  0.88 |
| TraesCS5D 02G566200 | gibberellin 20 oxidase | -3.57  -1.76 | -4.28  -1.29 | -4.14  -1.47 | -2.44  -1.56 | -2.7  -0.41 | -2.25  -0.63 | NA  -1.38 | NA  -1.5 |
| TraesCS1D 02G039400 | phenylalanine ammonia-lyase-like | NA  0.26 | NA  0.89 | -2.69  -0.83 | NA  0.51 | -4.21  -0.36 | -4.18  0.27 | -2.7  -0.96 | -3.59  0.05 |
| TRAESCS6A 02G303300 | pentatricopeptide repeat-containing protein PPR5 homolog. chloroplastic-like | -1.3  -2.51 | -1.34  -2.36 | -1.73  0.2 | -1.86  -0.39 | -2.69  -1.07 | -3.43  -0.61 | -3.56  -0.77 | -2.45  -0.09 |
| TRAESCS2A 02G496200 | molybdate transporter 2 | 1.15  0.54 | NA  1.46 | NA  0.68 | NA  -0.03 | 1.88  0.73 | 1.71  1.9 | 1.29  0.83 | 1.3  1.78 |
| TRAESCS6B 02G175100 | WRKY transcription factor | -2.57  -0.17 | -4.35  0.26 | -3.54  -0.59 | -1.96  0.85 | -4.55  0.86 | -3.44  0.4 | -3.71  -0.76 | -5.21  0.28 |
